# Chemical Stabilization and Retention-Predictive Modeling for Regiospecific Profiling of Labile Monoacylglycerols by UHPLC–HRMS

**DOI:** 10.1021/acs.analchem.6c05265

**Published:** 2026-10-10

**Authors:** Jun-Yi Zhou, Yan Zhang, Lu-Lu Wen, Yun Du, Wan-Cheng Zheng, Xiao-Xuan Duan, Miao Qu, Hai-Yu Zhao, Jian-Bo Wan

**Affiliations:** a State Key Laboratory of Mechanism and Quality of Chinese Medicine, 59193 University of Macau, Macau SAR 999078, China; b Institute of Chinese Medical Sciences, 59193 University of Macau, Macau SAR, 999078, China; c Shanxi Provincial Department-Municipal Key Laboratory Cultivation Base for Quality Enhancement and Utilization of Shangdang Chinese Medicinal Materials, School of Pharmacy, 74652 Changzhi Medical College, Changzhi, Shanxi 046000, China; d Department of Neurology, Xuanwu Hospital of Capital Medical University, Beijing 100053, China; e Institute of Chinese Materia Medica, 417459 China Academy of Chinese Medical Sciences, Beijing 100700, China

## Abstract

Regiospecific analysis of monoacylglycerols (MAGs) remains challenging because of their low endogenous abundance, the limited availability of regioisomeric standards, and rapid acyl migration between the *sn*-1 and *sn*-2 positions during sample handling. Here, we report an UHPLC–HRMS platform integrating chemical stabilization with retention-time prediction for confident profiling of MAG regioisomers in biological samples. Derivatization with 4-pyridineboronic acid (4-PyBA) selectively forms cyclic boronate esters with the glycerol *cis*-diol moiety, thereby suppressing acyl migration, enhancing electrospray ionization efficiency, and generating characteristic diagnostic ions. To minimize reliance on authentic standards, a three-dimensional quantitative structure–retention relationship (3D-QSRR) model was developed to predict chromatographic retention across structurally diverse MAGs. In parallel, a homologous series of fatty acid 1,2-pentanediol monoesters was synthesized as retention calibrants to correct retention time (RT) drift and improve method robustness. These components were integrated into a targeted parallel reaction monitoring (PRM) workflow supported by an in-house database of 288 annotated *sn*-1 and *sn*-2 MAGs. Application of this strategy to serum, brain, colon, and fecal samples from APP/PS1 mice and to serum samples from individuals with Alzheimer’s disease (AD) enabled the confident annotation of 118 endogenous MAGs, including 68 MAGs not currently catalogued in major public databases. Collectively, this integrated platform improves structural confidence and analytical robustness and establishes a transferable framework for regioisomer-resolved analysis of labile glycerolipids.

## Graphical abstract

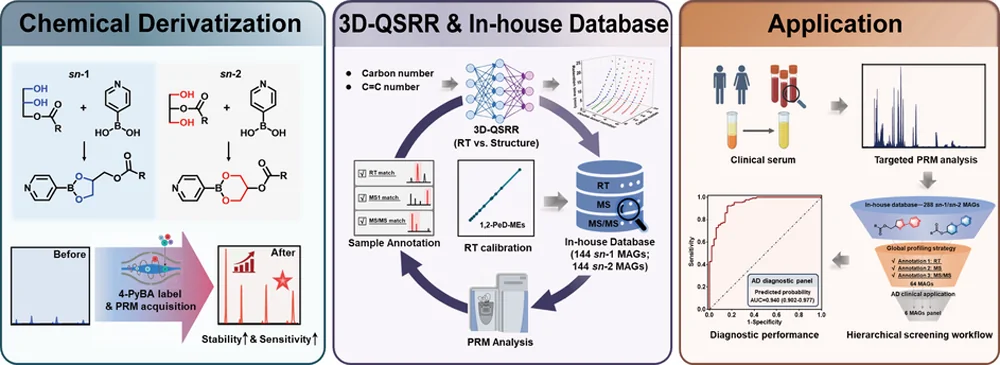

## Introduction

Monoacylglycerols (MAGs) are a structurally simple but biologically important class of glycerolipids involved in lipid signaling pathways, including the endocannabinoid system (ECS).^1^ Among them, 2-arachidonoylglycerol (2-AG) is a principal endogenous cannabinoid ligand, while other MAG species contribute to receptor signaling and lipid-mediated cellular regulation.^2^ Dysregulated MAG metabolism has been associated with neuroinflammation, synaptic dysfunction, and amyloid pathology in Alzheimer’s disease (AD),^3^ highlighting the biological significance of MAG regioisomers. Because *sn*-1 and *sn*-2 MAGs can exhibit distinct biochemical activities,^4^ accurate and regiospecific characterization of the MAG lipidome is essential for elucidating their functional roles in physiological and pathological processes.^5^ Nevertheless, comprehensive and confident profiling of endogenous MAGs in complex biological matrices remains analytically challenging.

Several intrinsic properties hinder reliable MAG analysis. Endogenous MAGs are generally present at low concentrations and are susceptible to ion suppression by more abundant lipid classes. Moreover, *sn*-1 and *sn*-2 regioisomers frequently coexist but are difficult to distinguish by conventional chromatographic and mass-spectrometric methods. Most critically, *sn*-2 MAGs readily undergo spontaneous acyl migration to the thermodynamically favored *sn*-1 form during sample preparation and chromatographic separation,^6,7^ resulting in structural ambiguity and quantitative bias. The limited availability of authentic regioisomeric standards further restricts reliable retention time (RT) assignment and structural confirmation across the chemically diverse MAG lipidome. Although gas chromatography–mass spectrometry (GC–MS),^8^ mass spectrometry imaging (MSI),^9^ and immunoassay-based methods^10^ have been explored, liquid chromatography–mass spectrometry (LC–MS) remains the most widely adopted platform because of its sensitivity and selectivity.^11^ However, conventional LC–MS workflows continue to be limited by acyl migration, low ionization efficiency, insufficient regioisomeric resolution, and substantial reliance on authentic standards for RT confirmation.

Chemical derivatization has been employed to improve MAG stability and MS response.^12,13^ Boronic acid reagents, such as 3-nitrophenylboronic acid (3-NPBA)^14^ and 2-methyl-4-phenylaminomethyl phenylboronic acid (2-methyl-4-PAMBA),^15^ selectively react with the *cis*-diol of the glycerol backbone to form cyclic boronate esters. Acyl chlorides, including benzoyl chloride^16^ and 3**-**(chlorosulfonyl)­benzoic acid,^17^ have also been used to derivatize the free hydroxyl group of MAGs. These derivatization strategies can enhance MS sensitivity, improve chromatographic separation, and in some cases, generate characteristic fragments. However, most reported methods have been demonstrated using only a limited number of MAG species. A systematic framework for comprehensive and confident regioisomer-specific profiling of structurally diverse MAGs therefore remains lacking.

Here, we report an integrated UHPLC–HRMS platform that combines stability-controlled derivatization with RT prediction for regiospecific profiling of MAGs. Derivatization with 4-pyridineboronic acid (4-PyBA) was employed to suppress acyl migration, enhance ionization efficiency, and generate diagnostic fragment ions. To reduce dependence on authentic standards, a three-dimensional quantitative structure–retention relationship (3D-QSRR) model was established to predict RTs across structurally diverse MAGs. In parallel, a homologous series of fatty acid 1,2-pentanediol monoesters (1,2-PeD-MEs) was synthesized via a one-pot enzymatic reaction and used as universal RT calibrants to correct chromatographic drift and improve interbatch reproducibility. Together, these components provide a systematic and transferable analytical framework for the confident annotation of labile MAG regioisomers in complex biological matrices.

## Materials and Methods

### Chemicals and Reagents

Thirty-eight MAG standards (Figures S1 and S2, Table S1) were obtained from Cayman Chemical (Ann Arbor, MI, USA). Sixteen fatty acid (FA) standards (Figure S3, Table S2) were purchased from NuChek Prep (Elysian, MN, USA). Owing to spontaneous acyl migration, most commercial MAG standards are supplied as mixtures of *sn*-1 and *sn*-2 isomers, enabling the acquisition of RT information for both regioisomers. Phenylboronic acid (PBA) and its derivatives, including 4-PyBA, 3-bromophenylboronic acid (3-BrPBA), 3-NPBA, and 3-(dimethylamino)­phenylboronic acid (3-DMAPBA), were purchased from Aladdin (Shanghai, China). Lipozyme 435 was obtained from Shanghai Zhili Biotechnology (Shanghai, China). Formic acid, ammonia, and trifluoroacetic acid (HPLC grade) were purchased from Sigma-Aldrich (St. Louis, MO, USA). Acetonitrile and methanol (MS grade) were obtained from Merck (Darmstadt, Germany). Ultrapure water was prepared using a Millipore purification system (Millipore, Bedford, MA). Stock solutions were stored at −20 °C until use.

### Synthesis of MAG Standards and RT Calibrants

Sixteen MAGs were synthesized via one-pot enzymatic esterification of free FAs with glycerol. The synthesized panel comprised saturated (6:0, 7:0, and 21:0–24:0), monounsaturated (13:1, 14:1, and 24:1), and polyunsaturated species (19:2, 21:2, 22:2, 22:3, 18:4, 20:5, and 22:5). Briefly, FA (1 mmol) and glycerol (16 mmol) were dissolved in tert-butanol (1 mL). Activated 4 Å molecular sieves (40 mg, activated at 120 °C) were added to remove residual moisture. After equilibration at 37 °C for 30 min, Lipozyme 435 (4%, *w*/*w*) was introduced, and the reaction was stirred at 350 rpm for 10 h. The reaction was terminated by dilution with acetonitrile before UHPLC**–**HRMS analysis.

For RT calibration, 1,2-PeD-MEs were synthesized under identical conditions using 10 FA standards (6:0–11:0, 13:1, 20:5, 22:4, and 22:6, Table S3) with 1,2-pentanediol. These homologous derivatives were used as universal RT calibrants to anchor retention prediction across batches.

### Human Serum Samples and Animal Samples

Human serum samples were collected from Xuanwu Hospital of Capital Medical University, including 130 patients with AD, 114 patients with mild cognitive impairment (MCI), and 50 age-matched healthy controls (HC). Clinical diagnoses were established according to the NINCDS-ADRDA criteria based on clinical and neuropsychological evaluations. Participants were included based on established clinical criteria for AD, MCI, or normal cognition, and were excluded if they presented with other neurological or psychiatric disorders, severe systemic diseases, or active infections that could confound serum profiling. The study protocol was approved by the Ethics Committee of Xuanwu Hospital, Capital Medical University (No. [2020] 097). Serum samples were stored at −80 °C until analysis.

Male APP/PS1 transgenic mice (C57BL/6J background, 12 months old) were obtained from Nanjing Junke Bioengineering (Nanjing, China). Animals were housed under specific pathogen-free conditions (23 ± 2 °C, 55 ± 5% humidity, 12 h light/dark cycle). Serum, brain, colon, and fecal samples were collected for analysis. All experimental procedures were approved by the Institutional Animal Care and Use Committee, Institute of Chinese Materia Medica, China Academy of Chinese Medical Sciences (No. 2025B150).

### Chemical Derivatization of MAGs with 4-PyBA

MAGs were extracted by liquid–liquid extraction as previously described with minor modifications.^18^ Briefly, serum (100 μL) was mixed with ice-cold ethyl acetate/hexane (9:1, *v*/*v*; 400 μL), ultrasonicated at 4 °C for 30 min, and incubated at −20 °C for 1 h. After centrifugation (12,000 rpm, 15 min), the organic phase was collected, evaporated under vacuum, and reconstituted in methanol (100 μL). Lipid extracts or MAG standards (1 μM, 100 μL) were reacted with 4-PyBA (10 mM in 50% methanol, 100 μL) and incubated at 4 °C (500 rpm, 1 h). After solvent evaporation, the residue was reconstituted in methanol (100 μL), centrifuged (12,000 rpm, 15 min, 4 °C), and subjected to UHPLC–HRMS.

### UHPLC–HRMS Analysis

MAG profiling was performed using a Vanquish UHPLC coupled to a Q-Exactive Plus Orbitrap mass spectrometer with a heated electrospray ionization (HESI) source (Thermo Fisher Scientific, Waltham, MA, USA). Chromatographic separation was achieved on an Acquity BEH C_18_ column (2.1 × 100 mm, 1.7 μm; Waters) at 40 °C using water (A) and acetonitrile (B) at 0.4 mL/min with the following gradient: 0–2 min, 70% B; 2–20 min, 70–100% B; 20–22 min, 100% B. Data were acquired in positive ion mode using targeted parallel reaction monitoring (PRM). HESI parameters were as follows: spray voltage, 3.5 kV; capillary temperature, 325 °C; auxiliary gas heater temperature, 300 °C; sheath gas/auxiliary gas, 40/10 arbitrary units; S-lens RF level, 55. PRM settings were: resolution 17,500 (FWHM), isolation window 1.0 Da, stepped normalized collision energy (NCE) 35/45/55%, automatic gain control (AGC) target 2 × 10^5^, maximum injection time 50 ms, and RT scheduling ± 0.5 min.

### Data Processing and Statistical Analysis

Raw data were processed using Xcalibur and TraceFinder (Thermo Fisher Scientific) for peak extraction and targeted quantification. The 3D-QSRR model was constructed in OriginPro 9.1 (OriginLab Corporation, Northampton, MA, USA), and multiple regression was performed using GraphPad Prism 8.0 (GraphPad Software, San Diego, CA, USA). Multivariate analysis was conducted in SIMCA 14.1 (Umetrics AB, Umeå, Sweden). Group differences were evaluated using two-tailed Student’s *t*-tests after normality assessment by the Kolmogorov–Smirnov test. A *p*-value of 0.05 was considered statistically significant.

## Results and Discussion

### Effects of pH, Temperature, and Incubation Time on MAG Isomerization

*sn*-2 MAGs readily undergo acyl migration to the thermodynamically favored *sn*-1 position, compromising regioisomer-specific analysis and quantification.^19,20^ To identify conditions that preserve MAG regiochemical integrity, acyl migration kinetics were systematically evaluated under different pH, temperature, and incubation time. Using *sn*-1 MAG (14:0) and *sn*-2 MAG (18:2) as representative standards, rapid interconversion occurred at pH ≥ 7.0, whereas both isomers remained stable under mildly acidic conditions (pH ≤ 6.0) (Figures 1A and 1B). Temperature markedly affected migration kinetics, with substantial isomerization occurring at 60 °C (Figures 1C and 1D). Time-dependent studies further revealed progressive conversion of *sn*-2 MAG (18:2) to its *sn*-1 isomer, as well as partial conversion of *sn*-1 MAG (14:0) to its *sn*-2 isomer after incubation at 60 °C for more than 2 h (Figures 1E and 1F). Collectively, these findings demonstrate that mildly acidic conditions, low temperatures, and short processing times are essential for minimizing acyl migration and preserving the native regiochemical composition of MAGs during sample preparation.

**Figure 1:**
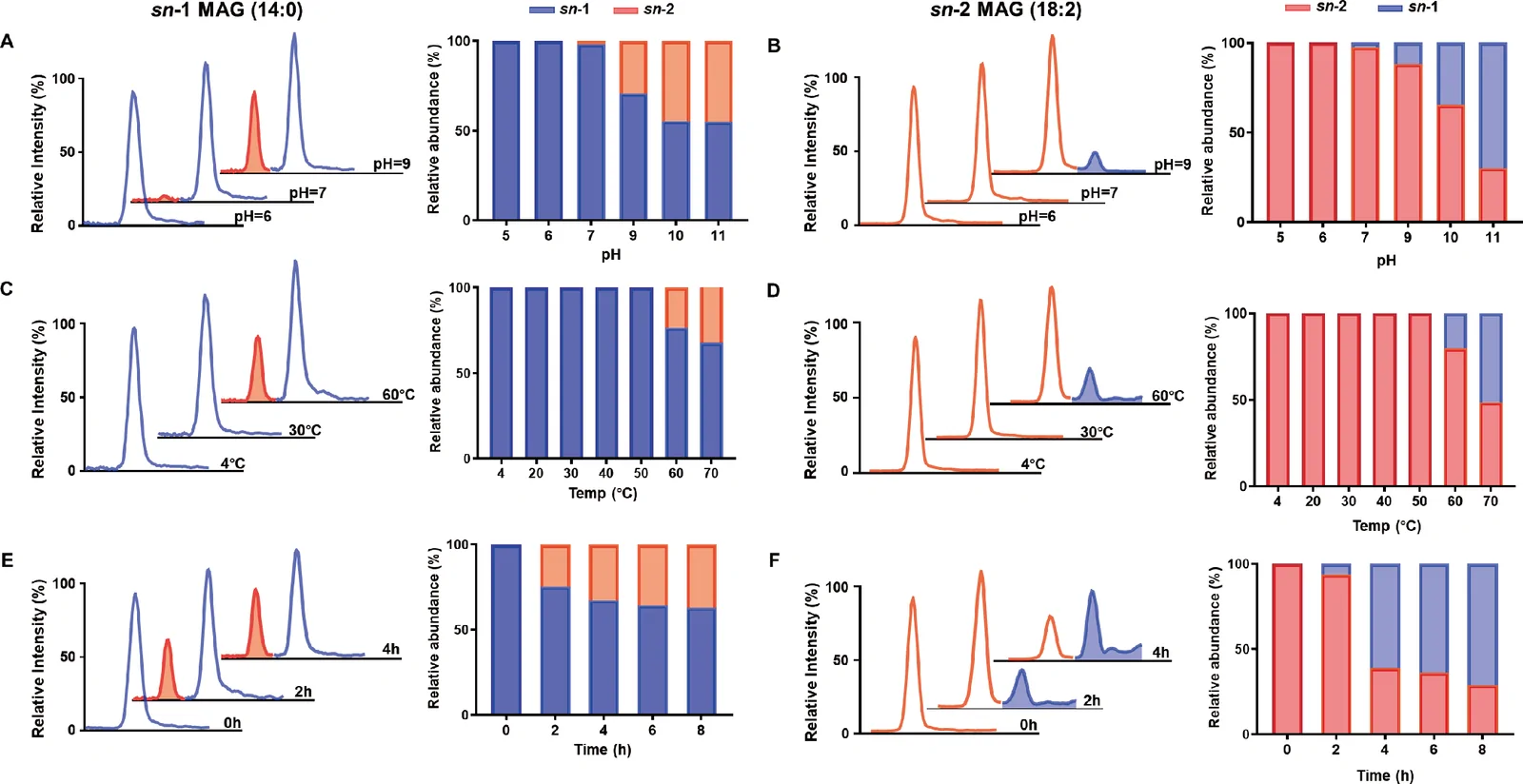
Effects of pH, temperature, and incubation time on MAG isomerization. (A, B) pH-dependent changes in (A) *sn*-1 MAG (14:0) and (B) *sn*-2 MAG (18:2). (C, D) Temperature effects after 2 h incubation. (E, F) Time-dependent changes at 60 °C.

### Optimization of 4-PyBA Derivatization Conditions

In addition to their intrinsic instability, endogenous MAGs are present at low abundance in biological samples and are highly susceptible to matrix-induced ion suppression, hindering their reliable reliable detection and structural characterization. To address these challenges, a boronic acid derivatization strategy was developed to simultaneously stabilize MAG regioisomers and enhance MS detectability. This approach exploits the *cis*-diol moiety of MAGs, which readily reacts with boronic acids to form cyclic boronate esters. To select the optimal derivatization reagent, several boronic acids, including PBA, 3-BrPBA, 3-NPBA, 3-DMAPBA, and 4-PyBA (Figure 2A), were systematically evaluated in terms of derivatization efficiency and signal enhancement. Among the tested reagents, 4-PyBA produced the highest MS signal intensities for both *sn*-1 and *sn*-2 MAGs, whereas analogues containing electron-withdrawing substituents resulted in lower MS responses (Figure 2B). Derivatization proceeds through rapid condensation between 4-PyBA and the MAG diol moiety under mild conditions (4 °C, 1 h), forming five- and six-membered cyclic boronate esters for *sn*-1 and *sn*-2 regioisomers, respectively (Figure 2C). This covalent locking of the glycerol backbone effectively stabilizes the MAG regioisomeric structures and suppresses acyl migration.

**Figure 2:**
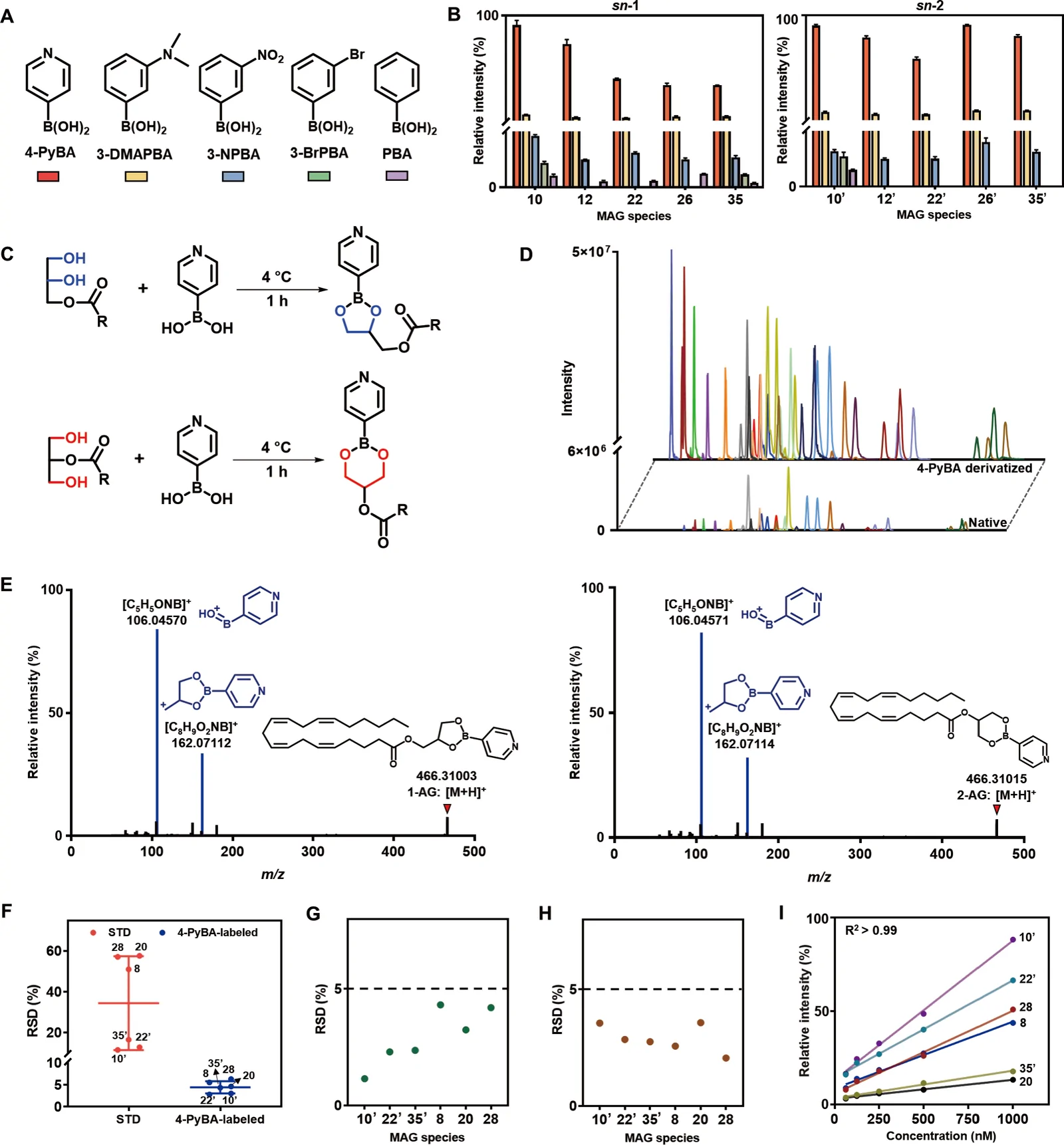
4-PyBA derivatization improves LC–MS performance and analytical robustness of MAGs. (A) Chemical structures of the five boronic acid reagents evaluated. (B) Relative MS signal intensities of representative *sn*-1 (left) and *sn*-2 (right) MAGs after derivatization. (C) Reaction scheme illustrating formation of five- and six-membered boronate esters from *sn*-1 and *sn*-2 MAGs with 4-PyBA. (D) Extracted ion chromatograms (EICs) of 22 *sn*-1/*sn*-2 MAG standards before and after 4-PyBA derivatization. (E) MS/MS spectra of 4-PyBA-derivatized 1-AG (left) and 2-AG (right). (F) Autosampler stability (7 °C, 72 h) of derivatized and underivatized MAGs. (G) Intraday precision (*n* = 5), (H) reproducibility (*n* = 3), and (I) calibration linearity of 4-PyBA-derivatized *sn*-1/*sn*-2 MAGs. MAG species numbering corresponds to Table S1.

To maximize derivatization efficiency, key reaction parameters were systematically optimized using single-factor experiments, including pH (5–11), temperature (4–60 °C), incubation time (10 min–6 h), and the MAG/4-PyBA molar ratio (1:500–1:10,000). Five representative pairs of *sn*-1/*sn*-2 MAG regioisomers (16:0, 18:0, 18:1, 18:2, and 20:4) were selected to evaluate derivatization performance. Optimal derivatization was achieved at pH 6.0, 4 °C for 1 h with agitation at 500 rpm using a MAG/4-PyBA molar ratio of 1:10,000 (Figure S4).

### Improvement of UHPLC–HRMS Performance upon 4-PyBA Derivatization

Derivatization with 4-PyBA markedly improved both the chromatographic and MS performance of MAGs. Extracted ion chromatograms of the derivatized MAGs exhibited substantially higher signal intensities than those of their native counterparts (Figure 2D). This enhancement was attributed to the introduction of a readily protonated pyridine moiety, which improves ionization efficiency in positive electrospray ionization mode. Collision-induced dissociation (CID) of 4-PyBA-labeled MAGs generated distinct and highly reproducible fragmentation patterns (Figure 2E). Although the *sn*-1 and *sn*-2 MAGs formed five- and six-membered boronate esters, respectively, both produced identical thermodynamically favored product ions. The characteristic base peak at *m*/*z* 106.04587 ([C_5_H_5_NOB]^+^), corresponding to the pyridylboronyl tag, and a secondary fragment at *m*/*z* 162.07209 ([C_8_H_9_O_2_NB]^+^) served as accurate-mass diagnostic ions. PRM monitoring of these fragments enabled sensitive targeted quantification and confident annotation of both known and theoretically predicted MAG species. By comparison, the MS/MS spectra of native MAGs were dominated by acylium ions generated following the neutral loss of glycerol (Figure S5), together with extensive nonspecific fragmentation in the low-mass range (*m*/*z* 50–200), thereby providing limited structural information. Furthermore, 4-PyBA-derivatized MAGs exhibited improved autosampler stability during storage at 7 °C for up to 72 h compared to underivatized standards (Figure 2F). Method validation also demonstrated high precision (Figure 2G), good reproducibility (Figure 2H), and excellent linearity (R^2^ > 0.99) over the tested concentration ranges (Figure 2I). Collectively, these results establish 4-PyBA derivatization as a sensitive, reproducible, and structurally informative strategy for quantitative profiling of trace MAGs in complex biological matrices.

### Construction of Quantitative Structure–Retention Relationship (QSRR) Predictive Model

Comprehensive structural annotation requires orthogonal chromatographic information; however, untargeted profiling of MAGs is constrained by the limited availability of authentic regioisomeric standards. To address this limitation, a one-pot enzymatic synthesis approach was implemented. Lipozyme 435–catalyzed esterification of glycerol with diverse FAs generated 16 additional *sn*-1/*sn*-2 MAG pairs (Figure 3A). Together with 22 commercially available standard pairs, a library of 38 MAG pairs was established (Table S1).

**Figure 3:**
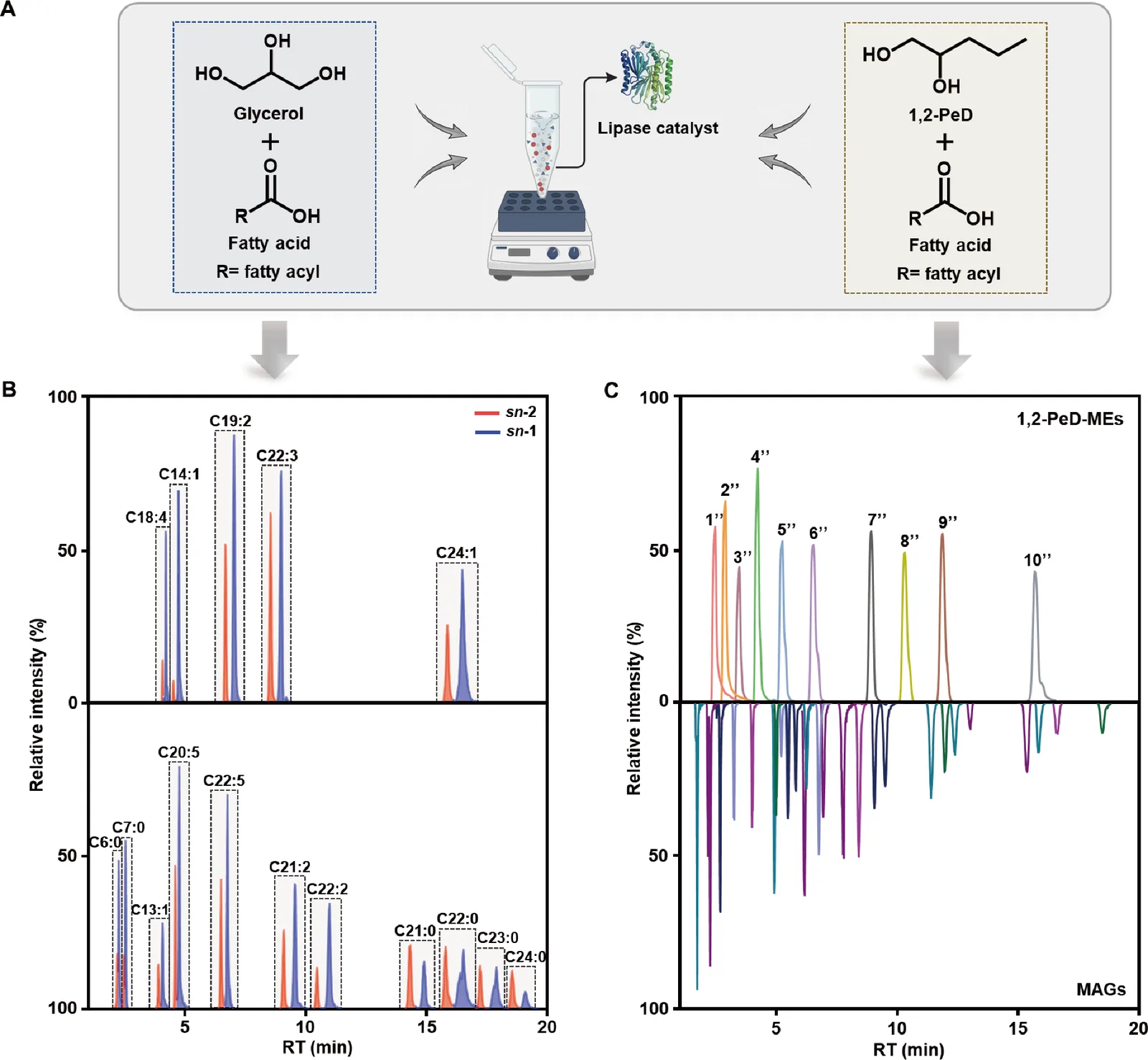
Synthesis of MAGs and retention time (RT) calibrants. (A) One-pot enzymatic synthesis of 16 MAG regioisomer pairs from fatty acids (FAs) and glycerol (left) and 10 RT calibrants from FAs and 1,2-pentanediol (1,2-PeD) (right). (B) Extracted ion chromatograms (EICs) of the synthesized MAG regioisomer pairs. (C) EICs of 1,2-PeD-MEs (top) and MAGs (bottom) after derivatization with 4-PyBA. Peaks 1″–10″ correspond to the RT calibrants listed in Table S3.

Based on this expanded reference set, QSRR models were constructed for 4-PyBA-derivatized MAGs. Notably, *sn*-2 MAGs consistently eluted earlier than their *sn*-1 counterparts (Figure 3B), likely owing to differences in polarity and stationary-phase interactions between the six- and five-membered boronate rings. For saturated MAGs ranging from 6:0 to 24:0, RT increased exponentially with acyl-chain carbon number for both regioisomers (R^2^ = 0.987 for *sn*-1; R^2^ = 0.989 for *sn*-2; Figures 4A and S6A). These strong correlations enabled the accurate RT prediction for MAGs lacking authentic standards, including MAG 9:0, 19:0, and 25:0 (Figures 4B and S6B). Similar exponential relationships were observed for monounsaturated and diunsaturated MAGs (Figure S7). An increasing unsaturation degree reduced hydrophobicity and shortened chromatographic retention. Within the C20 MAG series (20:0–20:5), RT was strongly and inversely correlated with the number of carbon–carbon double bonds (R^2^ = 0.993 for *sn*-1; R^2^ = 0.990 for *sn*-2; Figures 4C and S6C), enabling reliable prediction of highly unsaturated species such as MAG 20:6 (Figures 4D and S6D). Comparable trends were observed for the C18 and C22 series (Figure S8).

**Figure 4:**
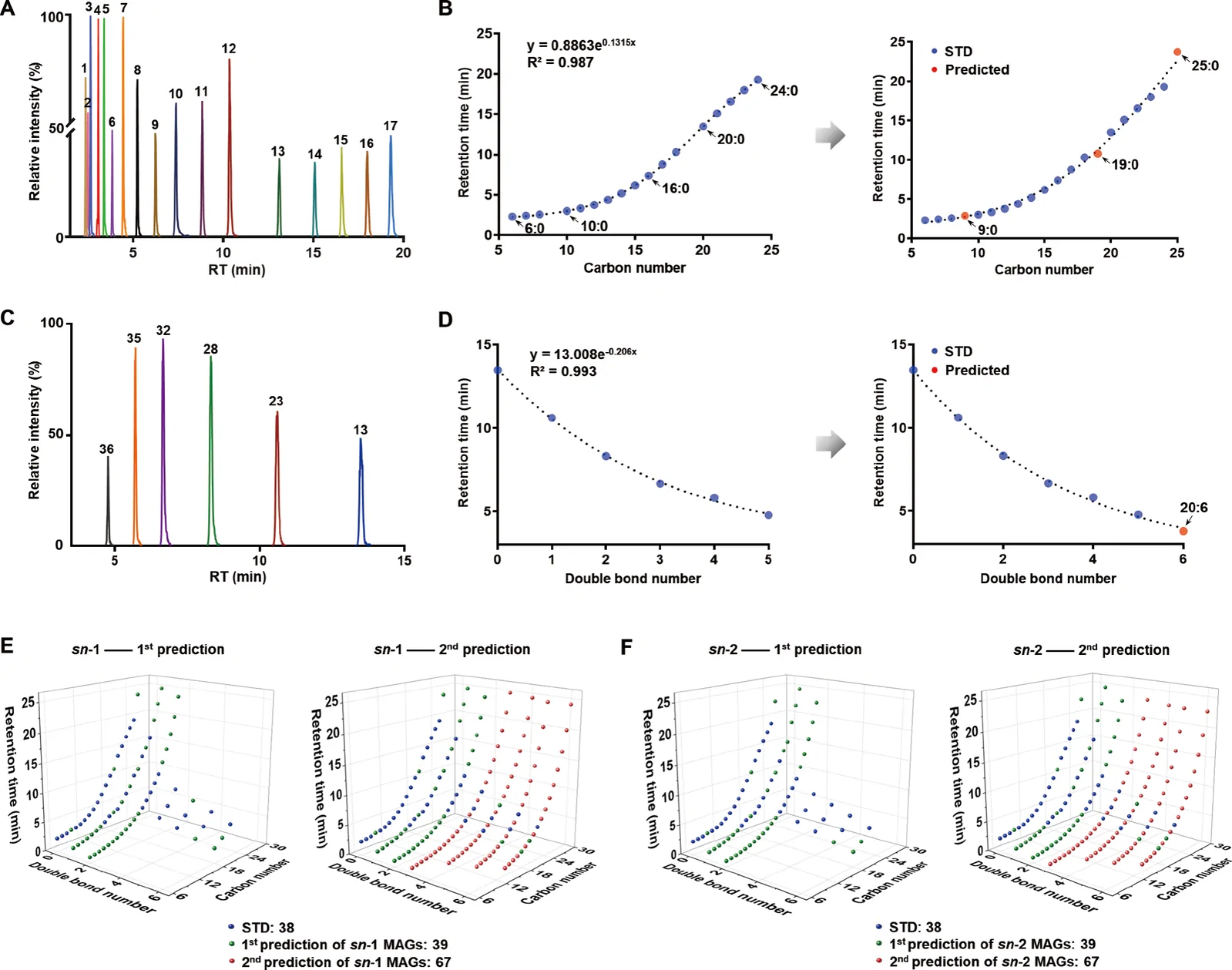
QSRR modeling and multidimensional RT prediction for MAGs. (A) Extracted ion chromatograms (EICs) of saturated *sn*-1 MAG standards after 4-PyBA derivatization (C6:0–C24:0). (B) Regression analysis correlating carbon number with RT (left) and two-dimensional (2D) RT prediction model for saturated MAGs (right; blue dots, reference standards; red dots, predicted species). (C) EICs of *sn*-1 C20 MAGs with different degrees of unsaturation. (D) Regression analysis plotted against the number of double bonds for *sn*-1 C20 MAGs (left) and corresponding 2D-RT prediction model (right). (E, F) Three-dimensional (3D) RT prediction models generated by multiple regression using carbon chain length and unsaturation degree for (E) *sn*-1 and (F) *sn*-2 MAGs, showing primary (right) and secondary (left) prediction surfaces. Peak numbering corresponds to those listed in Table S1. STD, standard.

To extend predictive coverage across the MAG lipidome, a 3D-QSRR model integrating acyl-chain carbon number and degree of unsaturation was developed. An iterative two-round strategy^21^ was employed. In the first round, experimentally determined RTs from 38 reference MAG pairs served as primary seeds, with at least three standards used to establish each predictive curve. These functions enabled the RTs of 39 intermediate species to be extrapolated. In the second round, predicted RTs were incorporated as secondary seeds to construct four additional predictive curves (Figure 4E,4F). This process ultimately enabled the prediction of RTs for 67 additional MAG species spanning C6–C30 acyl chains with 2–6 double bonds. The resulting 3D-QSRR framework combines predicted RTs with exact-mass and diagnostic MS/MS information, enabling annotation of 144 *sn*-1 and 144 *sn*-2 MAG species.

### Universal RT Calibration Using Synthesized 1,2-PeD-MEs

Because PRM-based identification relied on a narrow RT window (±0.5 min), precise RT alignment was essential. However, RT drift may arise from chromatographic and instrumental variability, including differences in chromatographic columns, mobile-phase compositions, and LC platforms. To correct such variability, a localized regression calibration method was applied.^22,23^ Although deuterated MAGs would be ideal internal standards, their high cost and limited availability preclude the construction of a continuous homologous series for RT calibration. Therefore, a homologous series of 1,2**-**PeD**-**MEs was synthesized via a one-pot enzymatic reaction (Figure 3A, Table S3) and used as universal RT calibrants. These compounds were selected because of their physicochemical similarity to MAGs and absence from biological matrices. Ten 1,2**-**PeD**-**MEs were evenly distributed across the chromatographic window of the targeted MAGs (Figure 3C). Method robustness was quantitatively evaluated across different gradient programs, columns, and LC platforms (Table S4), with RT accuracy defined as the ratio of predicted to actual RT. The calibration models exhibited excellent linearity (R^2^ > 0.999; Figure S9) and maintained RT accuracies within 94–110% under all tested conditions. These results demonstrate that 1,2-PeD-ME-based calibration effectively compensates for chromatographic drift, enabling reproducible and platform-independent identification of MAG regioisomers.

### Establishment of an In-house Database for MAG Recognition in Biological Samples

Leveraging the 3D-QSRR framework, we constructed a comprehensive in-house database containing 288 derivatized MAG species (144 *sn*-1 and 144 *sn*-2 isomers), encompassing C6–C30 acyl chains with 0–6 double bonds (Table S5). Each entry includes the exact monoisotopic mass, experimentally validated or predicted MS/MS information, and calibrated RT. Notably, only 50 of these species are currently included in major public databases, such as LIPID MAPS and HMDB, highlighting the expanded chemical coverage provided by our integrated strategy. For highly specific annotation, a targeted PRM workflow was implemented. Precursor isolation was performed using an isolation window of 1.0 Da, and fragmentation was restricted to scheduled RT windows of ±0.5 min, thereby improving the duty cycle and sensitivity for detecting trace-level MAGs. Confident annotation required both strict RT matching and the detection of characteristic pyridine–boronate diagnostic ions. Although 4*-*PyBA can potentially derivatize other *cis-*diol*-*containing molecules, such as saccharides and catecholamines, these highly hydrophilic species predominantly partition into the aqueous phase during the initial biphasic lipid extraction and are subsequently removed. Potential interferences can be further excluded through exact-mass filtering, distinct reversed-phase retention behavior, and highly specific MS/MS fragmentation patterns. This orthogonal filtering strategy ensures high analytical selectivity in complex biological matrices. To experimentally verify the exclusion of saccharide interference from targeted MAG profiling, 4-PyBA-derivatized glucose was analyzed and characterized under the established analytical conditions (Figure S10).

Ion mobility–mass spectrometry (IM–MS) provides rapid gas-phase separation and orthogonal collision cross-section (CCS) measurements for lipid profiling. However, conventional IM–MS often has limited ability to distinguish *sn-*1/*sn-*2 regioisomers because of their subtle CCS differences. Improved resolution may therefore require ultrahigh-resolution ion-mobility platforms or adduct-engineering approaches, such as metal-ion adduction.^24,25^ In contrast, our approach uses 4*-*PyBA derivatization to amplify the physicochemical differences between MAG regioisomers. By integrating these enhanced structural differences with 3D*-*QSRR modeling and 1,2*-*PeD*-*ME-based RT calibration, the proposed strategy enables baseline separation and reliable identification using standard, widely accessible UHPLC–HRMS platforms. This integrated chemical–computational hybrid approach therefore provides a robust and accessible complement to specialized IM–MS instrumentation for high-throughput analysis of MAG regioisomers.

### Global MAG Profiling in Biological Samples

Using 4-PyBA derivatization in conjunction with the in-house database, global MAG profiling was performed on the pooled samples from APP/PS1 mice (serum, brain, colon, and feces) and human AD serum (Figure 5A). Distinct matrix-specific compositional patterns were observed, with murine fecal samples exhibiting the greatest molecular diversity (Figure 5B). In total, 118 endogenous MAG species were confidently identified across the analyzed matrices, with *sn-*2 isomers detected more frequently than their *sn-*1 counterparts. The higher prevalence of *sn-*2 isomers is consistent with the regioselectivity of endogenous lipases. In mammalian lipid metabolism, enzymes such as diacylglycerol lipases (DAGL) and triacylglycerol lipases preferentially hydrolyze ester bonds at the *sn-*1 or *sn-*3 position, resulting in the formation of *sn-*2 MAGs as important metabolic intermediates and signaling molecules, including endocannabinoids.^26,27^ From an analytical perspective, *sn-*2 MAGs are thermodynamically unstable and susceptible to spontaneous acyl migration. Their abundant detection therefore supports the ability of rapid, low-temperature 4*-*PyBA derivatization to preserve the native regioisomeric state and minimize *sn-*2 to *sn-*1 acyl migration, which can confound conventional lipidomic analysis. Notably, 68 of the identified MAG species are not currently catalogued in LIPID MAPS or HMDB, substantially expanding the coverage of MAG species beyond existing public databases (Figure 5B, Table S6).

**Figure 5:**
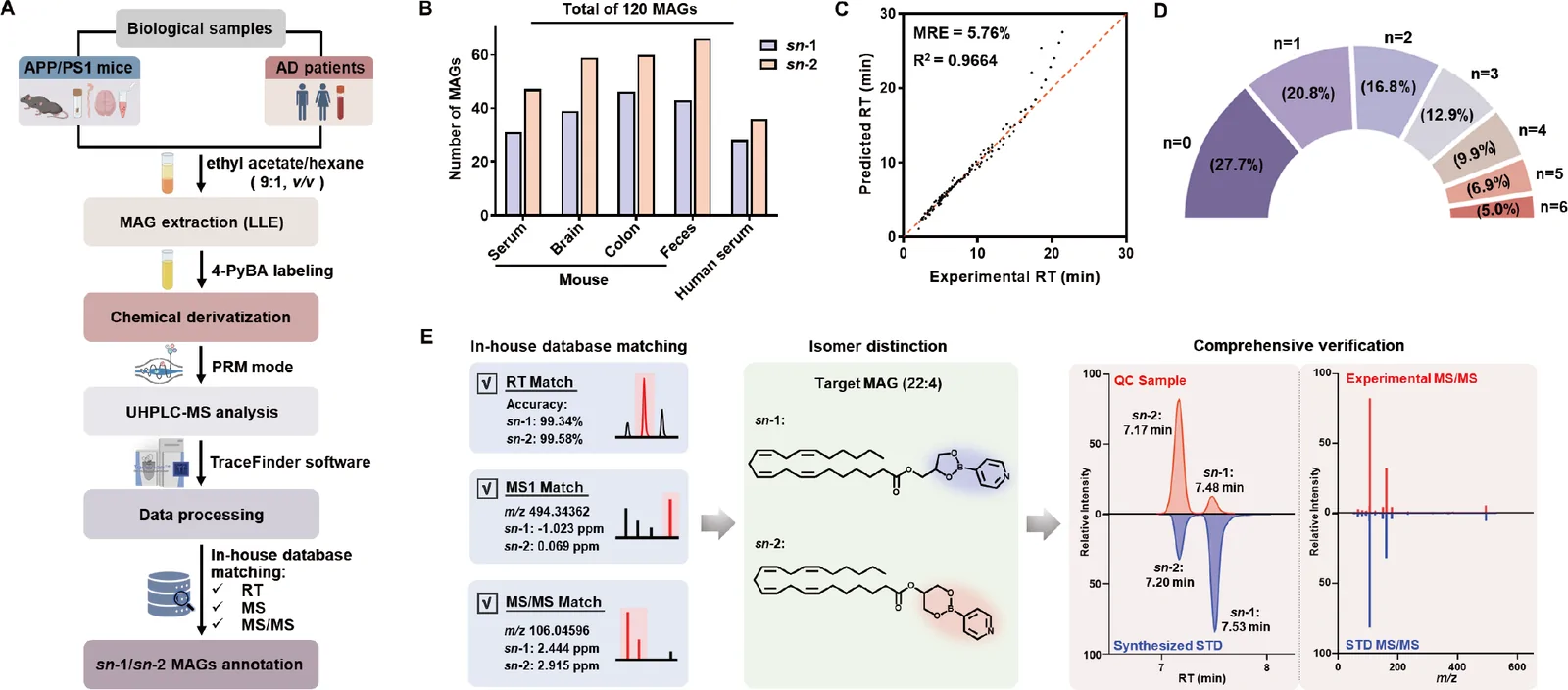
Global profiling of MAGs in biological samples using the integrated 4-PyBA derivatization strategy. (A) Workflow of the 4-PyBA derivatization platform enabling regiospecific MAG annotation in biological samples. (B) Distribution of identified *sn*-1 and *sn*-2 MAG species across biological samples. (C) Mean relative error (MRE) of QSRR-predicted RTs for 118 MAGs identified in biological samples. (D) Proportion of identified MAGs stratified by unsaturation degree. (E) Representative annotation workflow for *sn*-1/*sn*-2 MAG (22:4) in QC serum, integrating RT prediction, accurate mass measurement, and MS/MS spectral matching. Representative structural assignments were validated with synthesized standards, as demonstrated by mirror chromatograms and matched fragmentation patterns.

Predicted RTs showed strong agreement with experimental measurements (R^2^ = 0.9664), with a mean relative error (MRE) of 5.76% (Figure 5C), further validating the predictive performance of the QSRR model. The overall MAG composition was dominated by saturated (27.7%) and monounsaturated (20.8%) species (Figure 5D). Regioisomer assignments were established based on a stringent triple-criteria workflow integrating RT prediction, accurate mass measurement, and MS/MS spectral matching. For example, a precursor ion at *m*/*z* 494.34362 detected at RTs of 7.17 and 7.48 min in murine serum was assigned to *sn-*2 and *sn-*1 MAG (22:4), respectively, and subsequently confirmed by chemical synthesis (Figure 5E). Collectively, this integrated workflow enables confident regiospecific profiling of MAGs in complex biological matrices.

### Progressive Dysregulation of Serum MAGs in AD Patients

To evaluate the clinical applicability of MAG metabolic profiling in AD, the integrated analytical strategy was applied to human serum samples from HC, MCI, and AD cohorts. High analytical stability was demonstrated by the tight clustering of interspersed quality control (QC) samples relative to the widely dispersed serum samples in the principal component analysis (PCA) score plot (Figure S11A). Multivariate statistical analysis was performed to capture the MAG metabolic alterations across progressive stages of cognitive decline. Partial least-squares discriminant analysis (PLS-DA) revealed a progressive metabolic transition among the three groups (R^2^Y = 0.472, Q^2^ = 0.354, Figure 6A), with the MCI group positioned between the HC and AD groups, indicating stepwise remodeling of MAG metabolism across different stages of cognitive impairment.^28^ Pairwise OPLS-DA models further confirmed distinct metabolic separation at each clinical transition (HC vs MCI, Figure S11B; MCI vs AD, Figure S11C), with robustness validated by 200-iteration permutation testing (Figure S11D–F). To delineate the pathological alterations, an OPLS-DA model comparing HC and AD groups was constructed, yielding a reliable model (R^2^Y = 0.737, Q^2^ = 0.503, Figure 6B). Univariate analysis (volcano plot) identified 17 significantly dysregulated MAGs (FC > 2, VIP > 1 and *p* < 0.05) between the AD and HC groups (Figure 6C), with their relative abundance differences visualized by heatmap analysis (Figure 6D). Fold-change analyses across disease stages revealed that MAG perturbations were already evident between HC and MCI (Figure S11G) and became markedly exacerbated in end-stage AD (Figure S11H). Most MAGs, particularly the long-chain species (e.g., *sn-*1/*sn-*2 MAG (16:0), (18:0), and (20:0)), exhibited a progressive depletion from HC to MCI to AD.^29^

**Figure 6:**
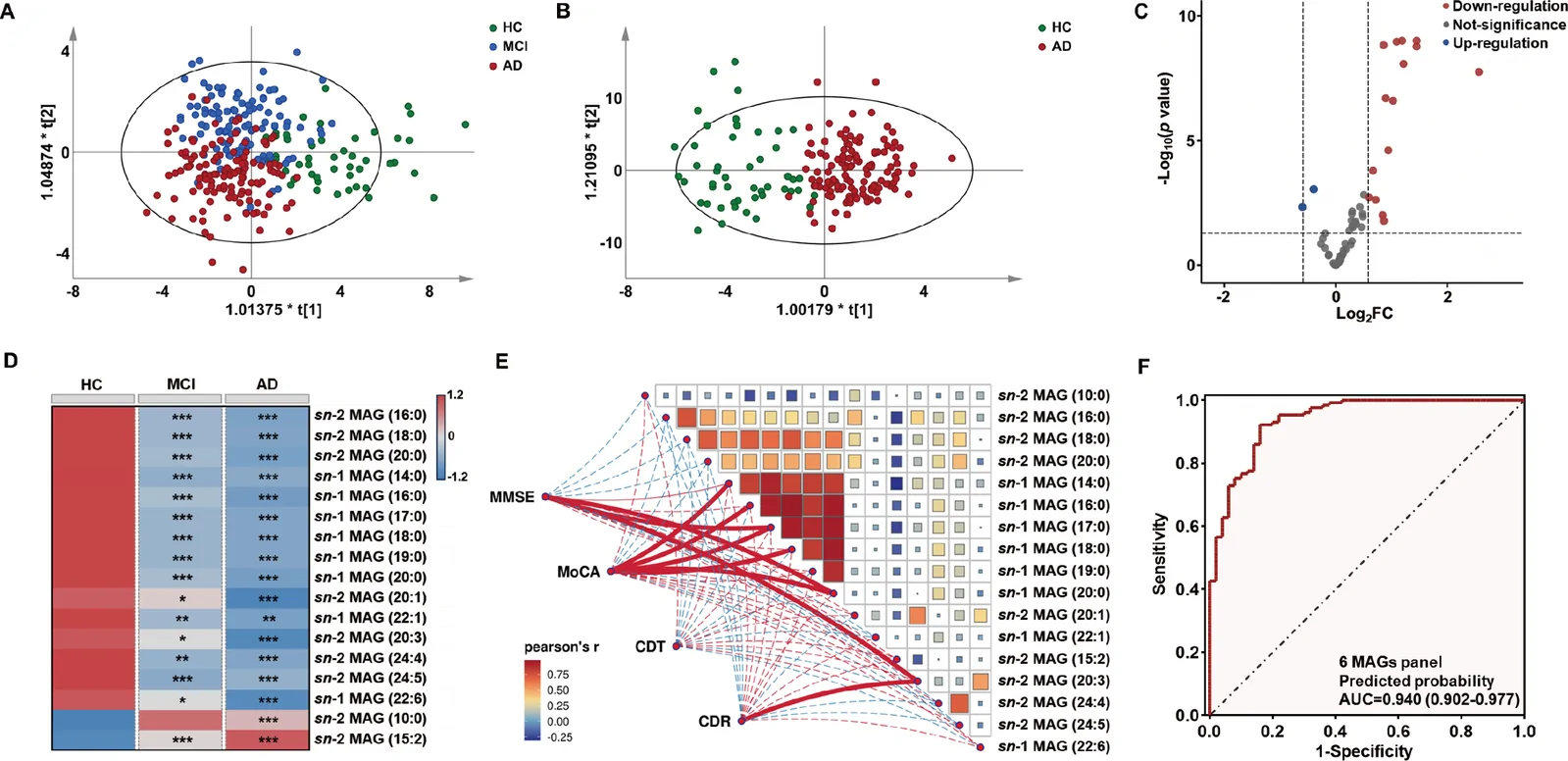
Metabolic profiling of MAG regioisomers and diagnostic evaluation in clinical serum. (A) PLS-DA score plot of healthy controls (HC, green), patients with mild cognitive impairment (MCI, blue), and patients with Alzheimer’s disease (AD, red). (B) OPLS-DA score plot showing the separation between HC and AD. (C) Volcano plot highlighting significantly dysregulated MAG species between AD and HC (blue dots, upregulated; red dots, downregulated; AD/HC ratio). (D) Heatmap of differentially regulated MAGs across clinical stages (* *p* < 0.05, ** *p* < 0.01, *** *p* < 0.001 vs HC group). (E) Bipartite correlation network linking cognitive scores, including Mini-Mental State Examination (MMSE), Montreal Cognitive Assessment (MoCA), Clock Drawing Test (CDT), and Clinical Dementia Rating (CDR), with dysregulated MAGs. The upper-right matrix shows intralipid Pearson correlations (color and size indicate direction and magnitude). Edges represent clinical–lipid associations (red, positive; blue, negative); line thickness reflects absolute correlation coefficients; solid lines indicate *p* < 0.05. (F) Combined ROC curve of six serum MAGs for AD discrimination.

Given that many MAGs function as precursors or signaling molecules within the ECS,^30^ their progressive reduction alongside cognitive decline suggests progressive disruption of endogenous neuroprotective lipid signaling during AD pathogenesis.^31^ In contrast, *sn-*2 MAG (10:0) and *sn-*2 MAG (15:2) were significantly elevated in AD. These divergent trends highlight the analytical necessity of chromatographic resolution at the regioisomer level to avoid masking potential disease-associated metabolic signatures.^32^

To contextualize the biological relevance of the dysregulated MAGs, a bipartite correlation network was constructed (Figure 6E). Within the MAG correlation matrix, a highly conserved coexpression module composed entirely of saturated *sn-*1 MAG isomers (MAG (14:0)–MAG (20:0), Pearson’s *r* > 0.5) was identified. When mapped to clinical indices, cognitive performance scales, including Mini-Mental State Examination (MMSE) and the Montreal Cognitive Assessment (MoCA), exhibited broad positive correlations with this specific *sn-*1 MAG cluster. Conversely, Clinical Dementia Rating (CDR) of dementia severity positively correlated exclusively with the polyunsaturated *sn-*2 MAG (20:3). Given these strong associations with cognitive decline, the diagnostic potential of representative differential MAGs was evaluated as a multiplexed biomarker panel. Receiver operating characteristic (ROC) analysis demonstrated that the combined serum MAG panel (*sn-*1 MAG (14:0), (16:0), (17:0), (18:0), (20:0), and *sn-*2 MAG (20:3)) achieved an AUC of 0.94 (95% CI: 0.902–0.977; Figure 6F), indicating high discriminatory performance. This multimolecular signature mitigates individual biological variability and demonstrates potential as a clinically informative tool for early evaluation of cognitive decline and AD progression.^33,34^

To explore the potential biological relevance of these MAGs, molecular docking was performed against eight key endocannabinoid-related receptors (Figure S12).^35,36^ Strong binding affinities were observed toward PPARα/γ, TRPV1, and GPR119. Notably, *sn*-2 MAG (20:3), which uniquely correlated with dementia severity, adopted a favorable binding pose within the GPR119 pocket, forming hydrogen bonds (∼1.9 Å) with Glu261 and Phe157, while its polyunsaturated acyl chain interacted with Trp265, Ala177, and Val93 through hydrophobic interactions. This spatial complementarity critically depends on the *sn*-2 configuration. These findings provide mechanistic support for the distinct clinical behavior of *sn*-2 MAG (20:3) and underscore the necessity of isomer-level resolution for identifying AD-related lipid biomarkers. Further studies are required to clarify its precise biological role in AD pathogenesis.

## Conclusions

In summary, we developed an integrated UHPLC–HRMS platform combining chemical stabilization and RT prediction for comprehensive regioisomeric MAG profiling. The platform employs 4-PyBA derivatization to suppress acyl migration, enhance ionization efficiency, and generate characteristic diagnostic ions. To minimize reliance on limited availability of authentic standards, a 3D-QSRR predictive model was integrated with synthesized 1,2-PeD-ME retention calibrants, enabling accurate RT prediction and improved analytical robustness. Using the resulting targeted PRM workflow, 118 endogenous MAGs, including 68 not currently catalogued in major public databases, were annotated in tissues and biofluids from APP/PS1 mice and in human AD serum. A serum panel comprising *sn*-1 MAG (14:0), (16:0), (17:0), (18:0), (20:0), and *sn*-2 MAG (20:3) showed potential as a diagnostic signature for AD. Collectively, this platform provides a robust and transferable approach for regioisomer-resolved analysis of labile glycerolipids, facilitating deeper insights into MAG metabolism and its potential roles in the pathogenesis and progression of diseases.

## Supplementary Material



## Notes

Chemical structures of 38 *sn*-1 and 38 *sn*-2 MAG reference standards, chemical structures of 16 fatty acid standards, optimization of 4-PyBA derivatization conditions with *sn*-1 MAGs and *sn*-2 MAGs, MS/MS spectra of native 2-AG and 1-AG standards, quantitative structure–retention relationship (QSRR) modeling for *sn*-2 MAGs (*n* = 0 and C20 series), QSRR modeling for unsaturated MAGs (*n* = 1 and *n* = 2), QSRR modeling of MAGs with acyl carbon numbers of 18 (C18 series) and 22 (C22 series), regression analysis and calibration performance of retention times (RTs) for 1,2-PeD-MEs under different chromatographic conditions, serum MAG profiles in healthy controls (HC), mild cognitive impairment (MCI), and Alzheimer’s disease (AD), molecular docking of six diagnostic MAG biomarkers with eight related receptors, detailed information on 38 MAG regioisomer pairs (reference standards) and their corresponding 4-PyBA-labeled derivatives, detailed information on 16 FA standards used for MAG synthesis, ten 1,2-PeD-MEs used for the RT calibration, LC gradient programs, columns, and instrument systems used in RT calibration experiments, detailed information on the in-house database of *sn*-1 and *sn*-2 MAGs, detailed information on 118 *sn*-1 and *sn*-2 MAGs identified in biological samples (PDF)
