# Supplementary material for: Chemical Stabilization and Retention-Predictive Modeling for Regiospecific Profiling of Labile Monoacylglycerols by UHPLC–HRMS
Source: Anal Chem. 2026 Oct 10;98(43):29991–30001. doi: 10.1021/acs.analchem.6c05265 (PMC13737999; doi:10.1021/acs.analchem.6c05265)
Supplement: Supplementary file 1 [file ac6c05265_si_001.pdf]

Supporting Information

Chemical Stabilization and Retention-Predictive Modeling for Regiospecific Profiling of Labile Monoacylglycerols by UHPLC–HRMS

Jun-Yi Zhou[a,b], Yan Zhang[c], Lu-Lu Wen[d], Yun Du[d], Wan-Cheng Zheng[d], Xiao-Xuan Duan[d], Miao Qu[d,*], Hai-Yu Zhao[e,*], Jian-Bo Wan[a,b,*]

[a] State Key Laboratory of Mechanism and Quality of Chinese Medicine, University of Macau, Macau SAR, 999078, China

[b] Institute of Chinese Medical Sciences, University of Macau, Macau SAR, 999078, China

[c] Shanxi Provincial Department-Municipal Key Laboratory Cultivation Base for Quality Enhancement and Utilization of Shangdang Chinese Medicinal Materials, School of Pharmacy, Changzhi Medical College, Changzhi, Shanxi, 046000, China.

[d] Department of Neurology, Xuanwu Hospital of Capital Medical University, Beijing, 100053, China.

[e] Institute of Chinese Materia Medica, China Academy of Chinese Medical Sciences, Beijing, 100700, China

***Corresponding author**

Prof. Jian-Bo Wan,

Email: jbwan@um.edu.mo

Institute of Chinese Medical Sciences, University of Macau,

Avenida da Universidade, Taipa, Macao, China

Prof. Hai-yu Zhao

Email: hyzhao@icmm.ac.cn

Institute of Chinese Materia Medica, China Academy of Chinese Medical Sciences, Beijing, 100700, China

Prof. Miao Qu

Email: qumiao@xwhosp.org

Department of Neurology, Xuanwu Hospital of Capital Medical University, Beijing, 100053, China.

Supporting Information

Table of contents

Figure S1. Chemical structures of 38 *sn*-1 MAG reference standards..S3

Figure S2. Chemical structures of 38 *sn*-2 MAG reference standards... S4

Figure S3. Chemical structures of 16 fatty acid standards...S5

Figure S4. Optimization of 4-PyBA derivatization conditions for *sn*-1 and *sn*-2 MAGs..................S6

Figure S5. MS/MS spectra of native (A) 2-AG and (B) 1-AG standards...S7

Figure S6. QSRR modeling for *sn*-2 MAGs (n = 0 and C20 series)..S7

Figure S7. QSRR modeling for unsaturated MAGs (n = 1 and n = 2)..S8

Figure S8. QSRR modeling of MAGs with acyl carbon numbers of 18 (C18 series) and 22 (C22 series) S9

Figure S9. Regression analysis and calibration performance of retention times (RTs) for 1,2 PeD MEs under different chromatographic conditions S10

Figure S10. EIC and MS/MS spectrum of the 4-PyBA derivatized glucose.............................S11

Figure S11. Serum MAG profiles in healthy controls (HC), mild cognitive impairment (MCI), and Alzheimer's disease (AD) S12

Figure S12. Molecular docking of six diagnostic MAG biomarkers with eight related receptors ...S13

Table S1. Detailed information on 38 MAG regioisomer pairs (reference standards) and their corresponding 4-PyBA-labeled derivatives S14

Table S2. Detailed information on 16 fatty acid standards used for MAG synthesis S16

Table S3. Ten 1,2-PeD-MEs used for the RT calibration S17

Table S4. LC gradient programs, columns, and instrument systems used in RT calibration experiments S17

Table S5. Detailed information on the in-house database of *sn*-1 and *sn*-2 MAGs S18

Table S6. Detailed information on 118 *sn*-1 and *sn*-2 MAGs identified in biological samples........S21

Supporting Information

Supplementary Figures

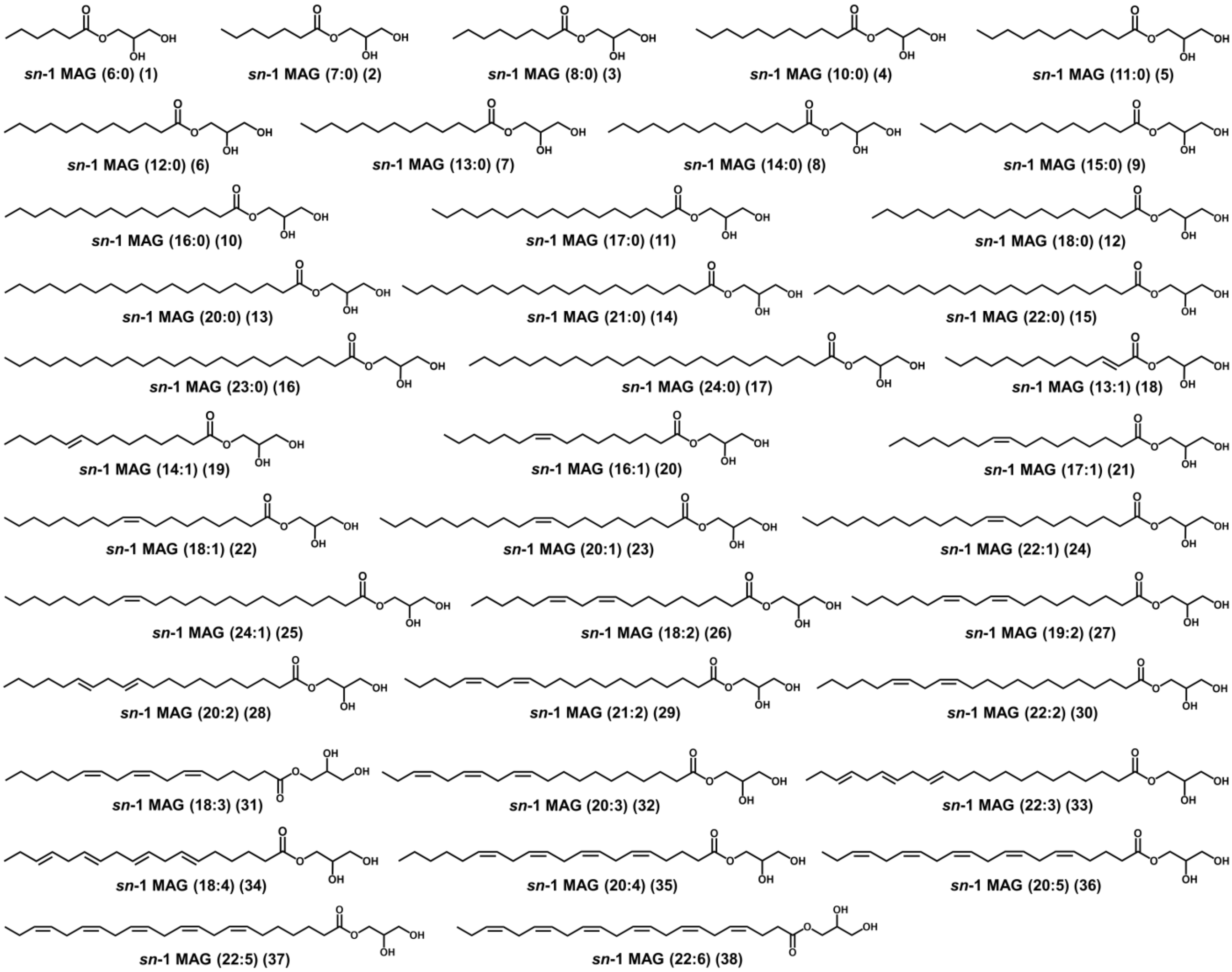

Figure S1. Chemical structures of 38 *sn*-1 MAG reference standards.

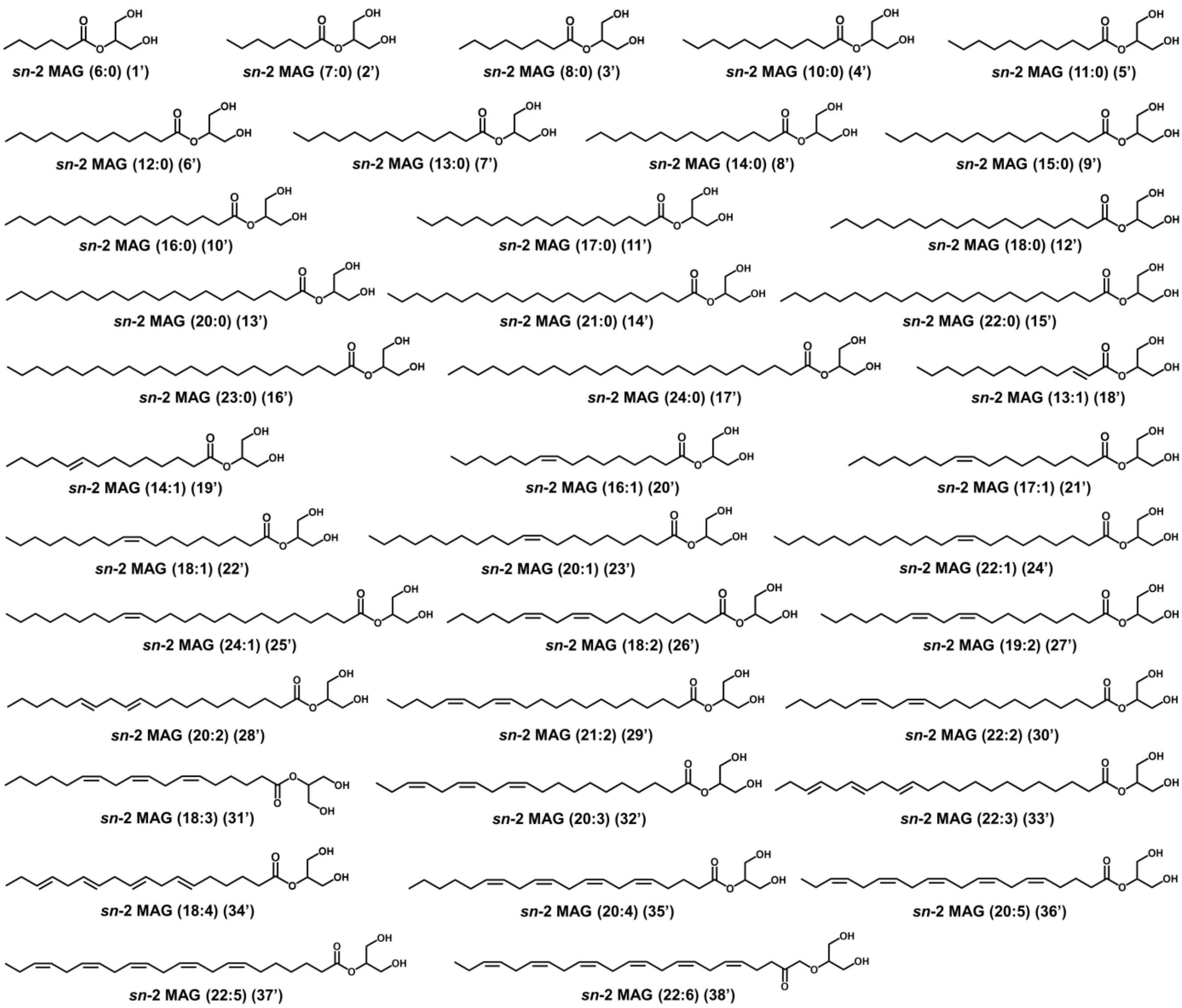

Figure S2. Chemical structures of 38 *sn*-2 MAG reference standards.

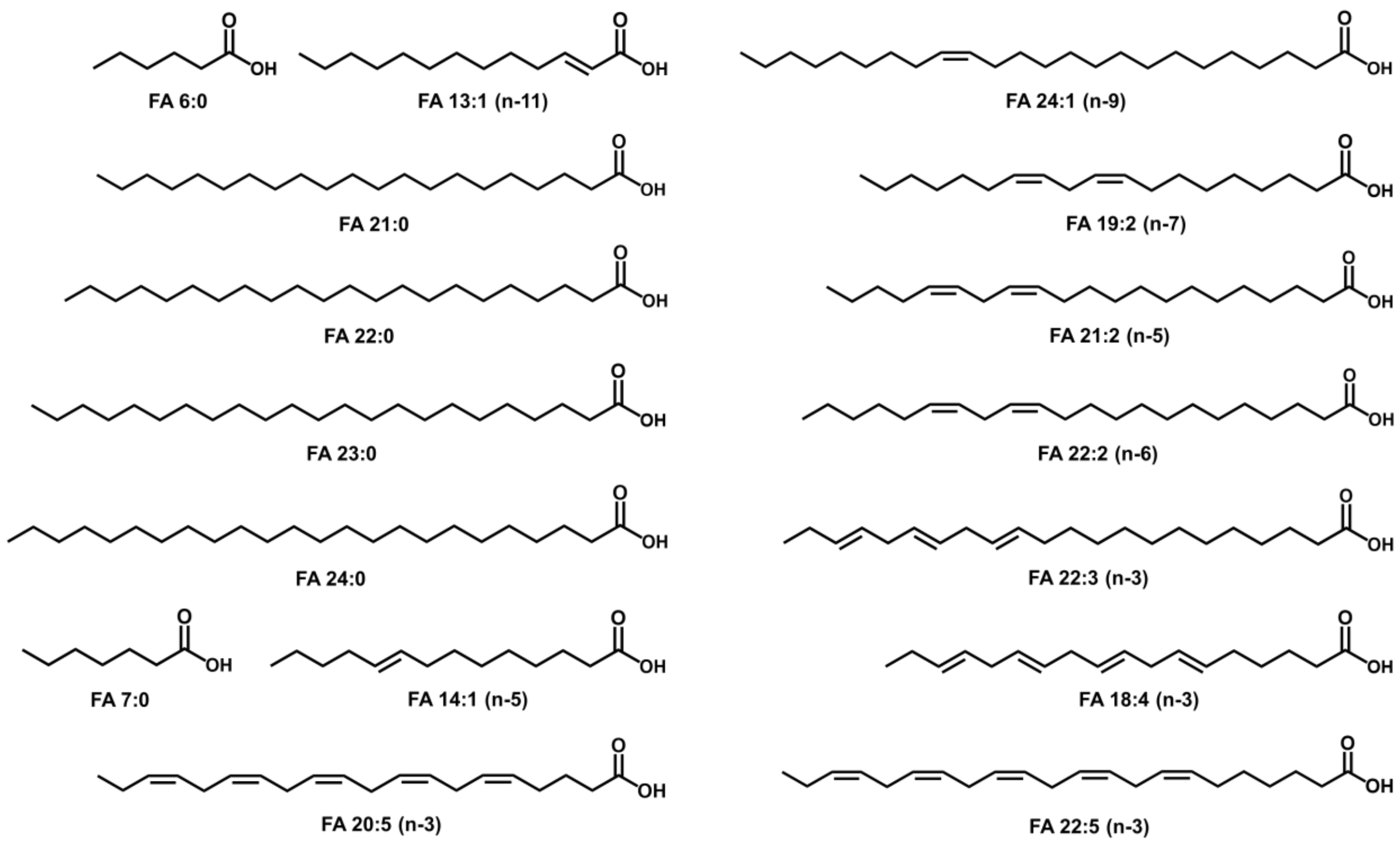

Figure S3. Chemical structures of 16 fatty acid standards.

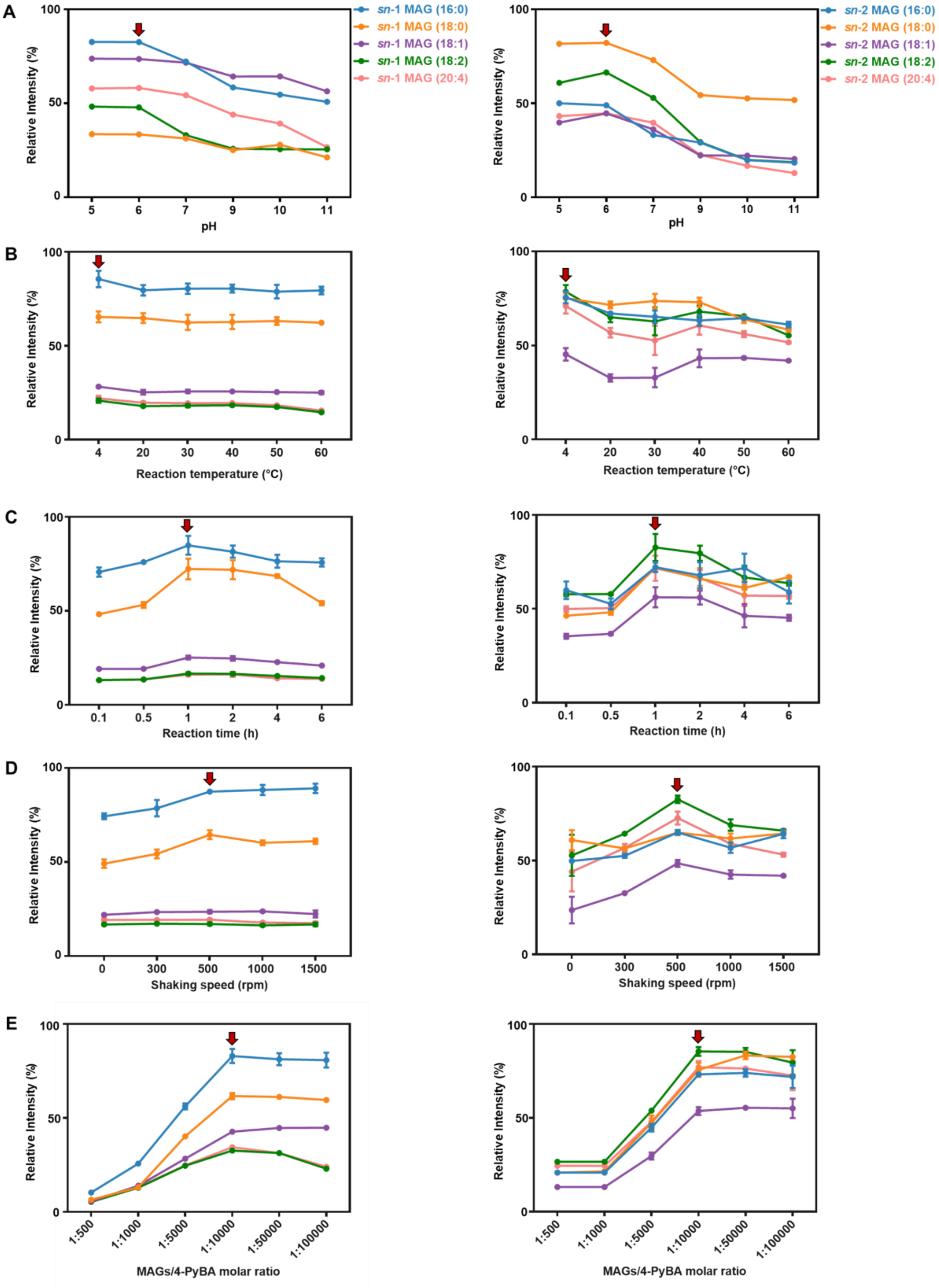

Figure S4. Optimization of 4-PyBA derivatization conditions for *sn*-1 (left) and *sn*-2 (right) MAGs. Effects of (**A**) pH, (**B**) reaction temperature, (**C**) reaction time, (**D**) shaking speed, and (**E**) MAGs/4-PyBA molar ratio.

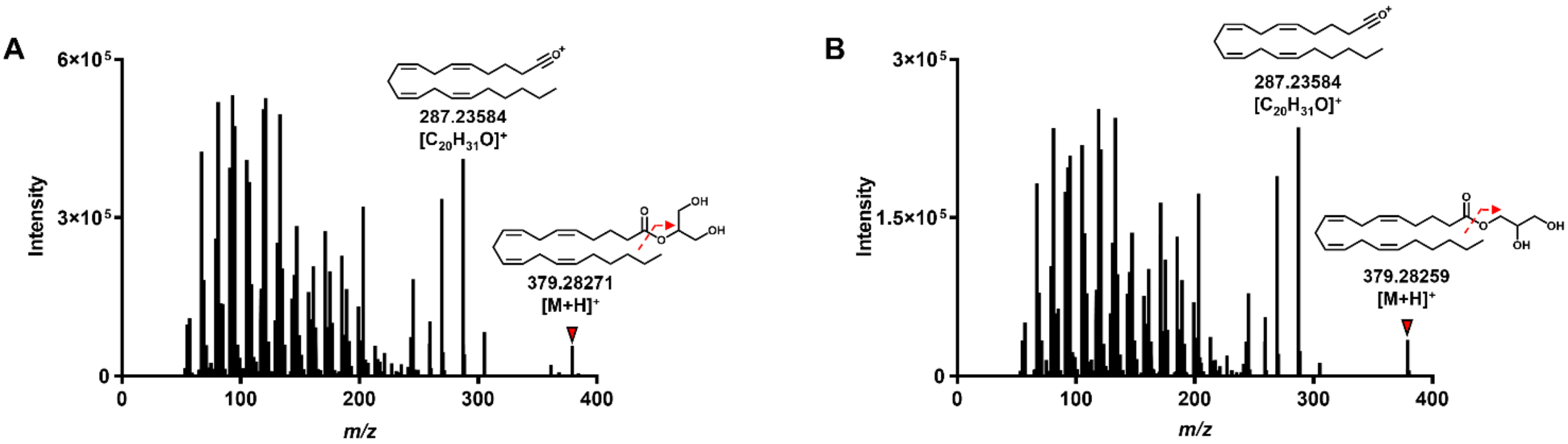

Figure S5. MS/MS spectra of native (**A**) 2-AG and (**B**) 1-AG standards.

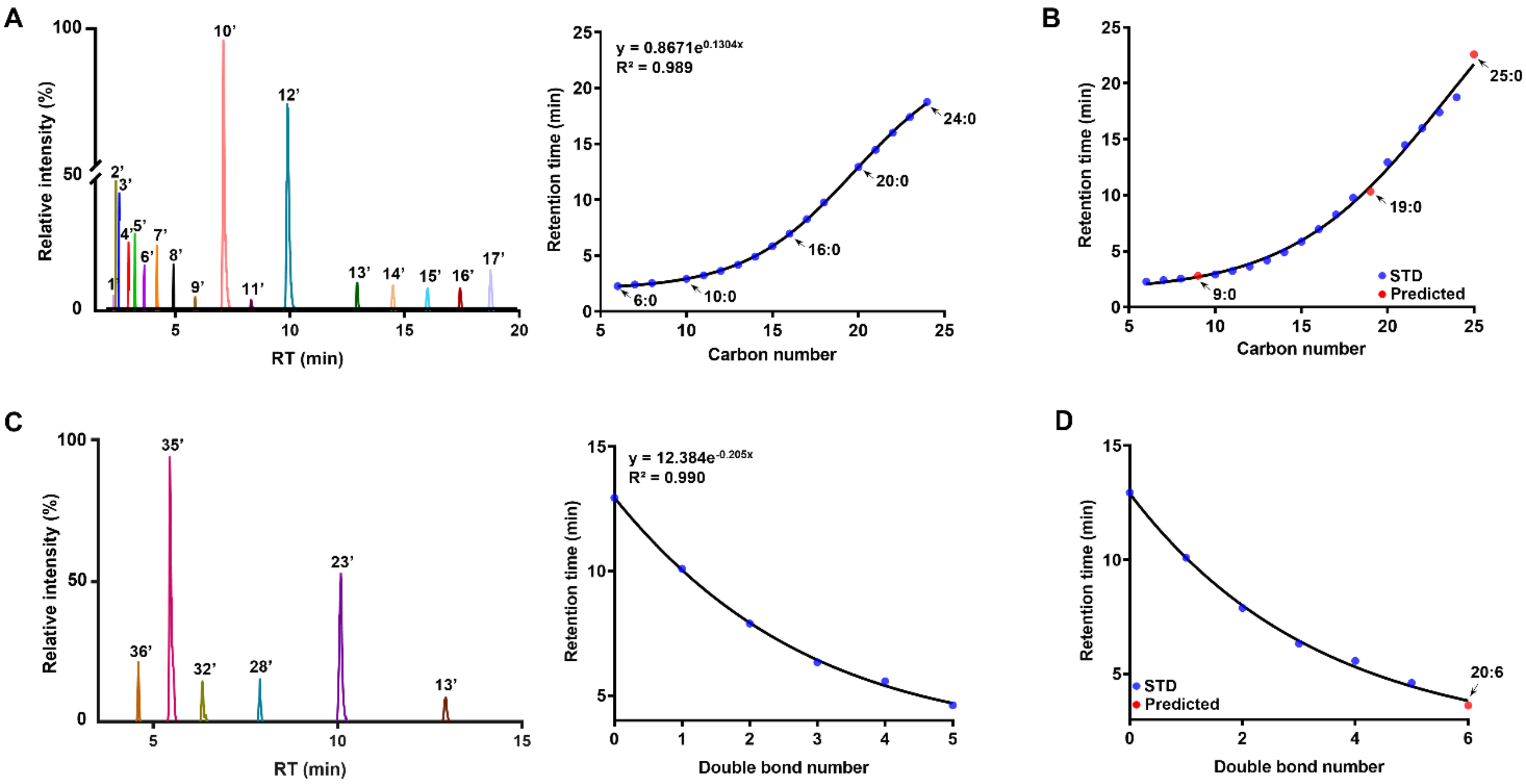

Figure S6. Quantitative structure-retention relationship (QSRR) modeling for *sn*-2 MAGs (n = 0 and C20 series). (**A**) Extracted ion chromatograms (EICs, left) of 4-PyBA-derivatized saturated *sn*-2 MAGs (n=0, 6:0–24:0), and regression analysis (right) correlating carbon number with retention time (RT). (**B**) Two-dimensional (2D) RT prediction model for saturated MAGs (blue dots, reference standards; red dots, predicted species). (**C**) EICs (left) and RT regression (right) plotted against the number of double bonds for *sn*-2 C20 MAGs. (D) 2D RT prediction model for C20 MAGs based on unsaturation degree (blue dots, reference standards; red dot, predicted species). 36’, *sn*-2 MAG (20:5); 35’, *sn*-2 MAG (20:4); 32’, *sn*-2 MAG (20:3); 28’, *sn*-2 MAG (20:2); 23’, *sn*-2 MAG (20:1); and 13’, *sn*-2 MAG (20:0). Peak numbering corresponds to **Table S1**.

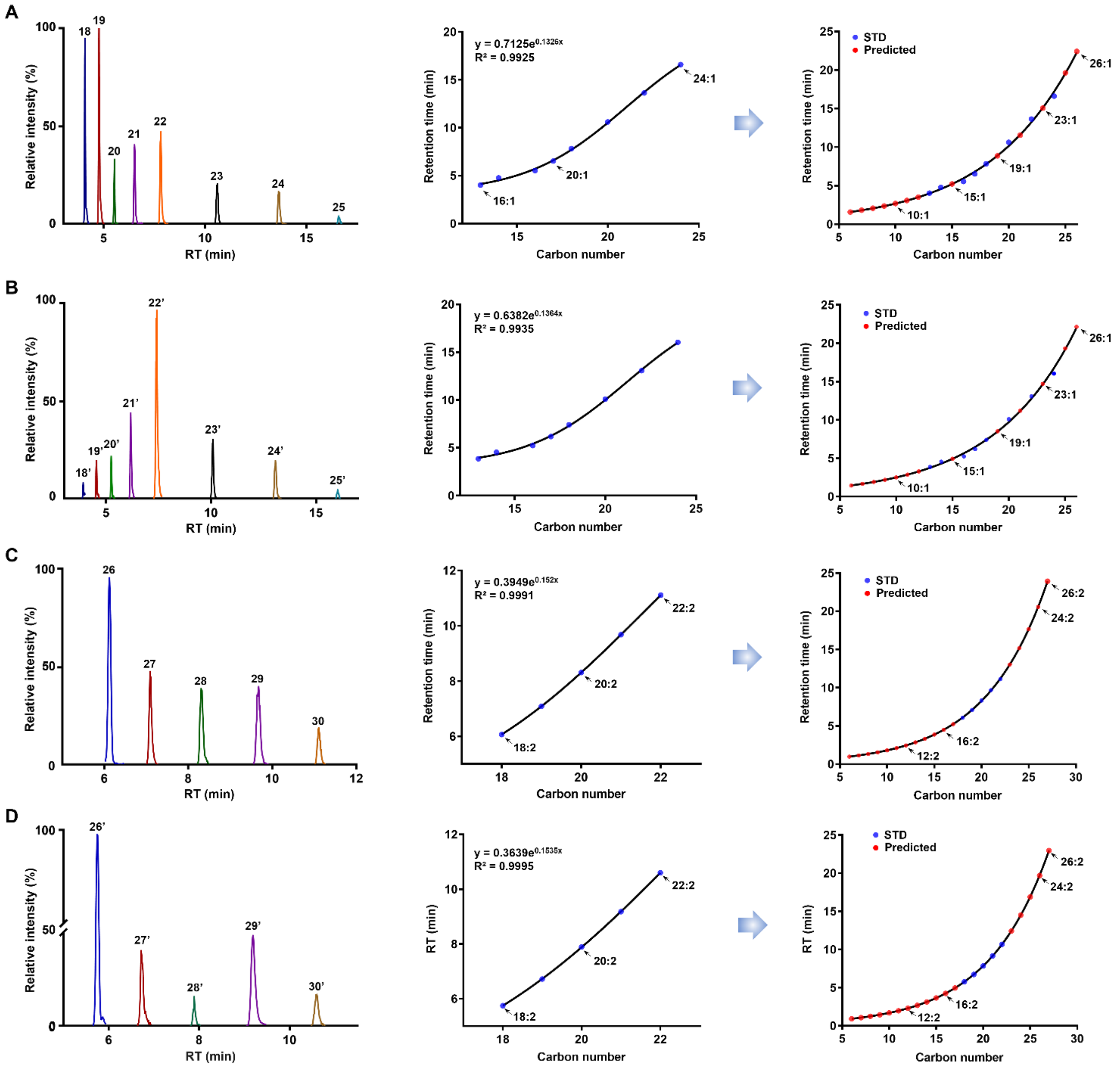

Figure S7. Quantitative structure-retention relationship (QSRR) modeling for unsaturated MAGs (n = 1 and n = 2). Each panel shows EICs (left), RT–carbon number regression (middle), and corresponding 2D RT prediction models (right). Monounsaturated MAGs (n = 1) with an acyl chain at (**A**) *sn*-1 and (**B**) *sn*-2. Diunsaturated MAGs (n = 2) at (**C**) *sn*-1 and (**D**) *sn*-2. Blue dots, reference standards; red dot, predicted species. Peak numbering corresponds to **Table S1**.

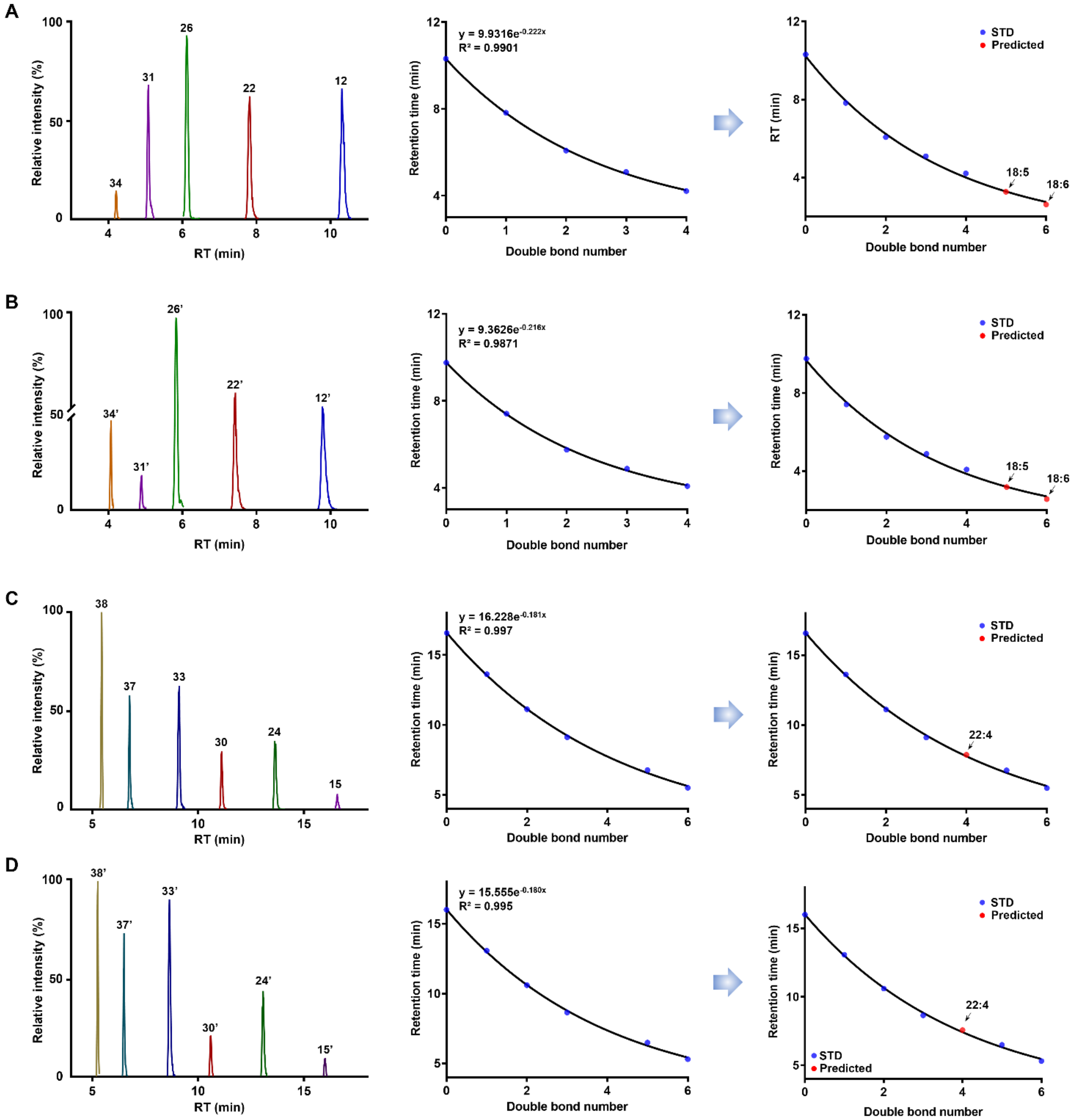

Figure S8. Quantitative structure-retention relationship (QSRR) modeling of MAGs with acyl carbon numbers of 18 (C18 series) and 22 (C22 series). Each panel shows EICs (left), RT-double bond number regression (middle), and corresponding 2D RT prediction models (right). (**A**) *sn*-1 C18 MAGs; (**B**) *sn*-2 C18 MAGs; (**C**) *sn*-1 C22 MAGs; (**D**) *sn*-2 C22 MAGs. Blue dots, reference standards; red dot, predicted species. Peak numbering corresponds to **Table S1**.

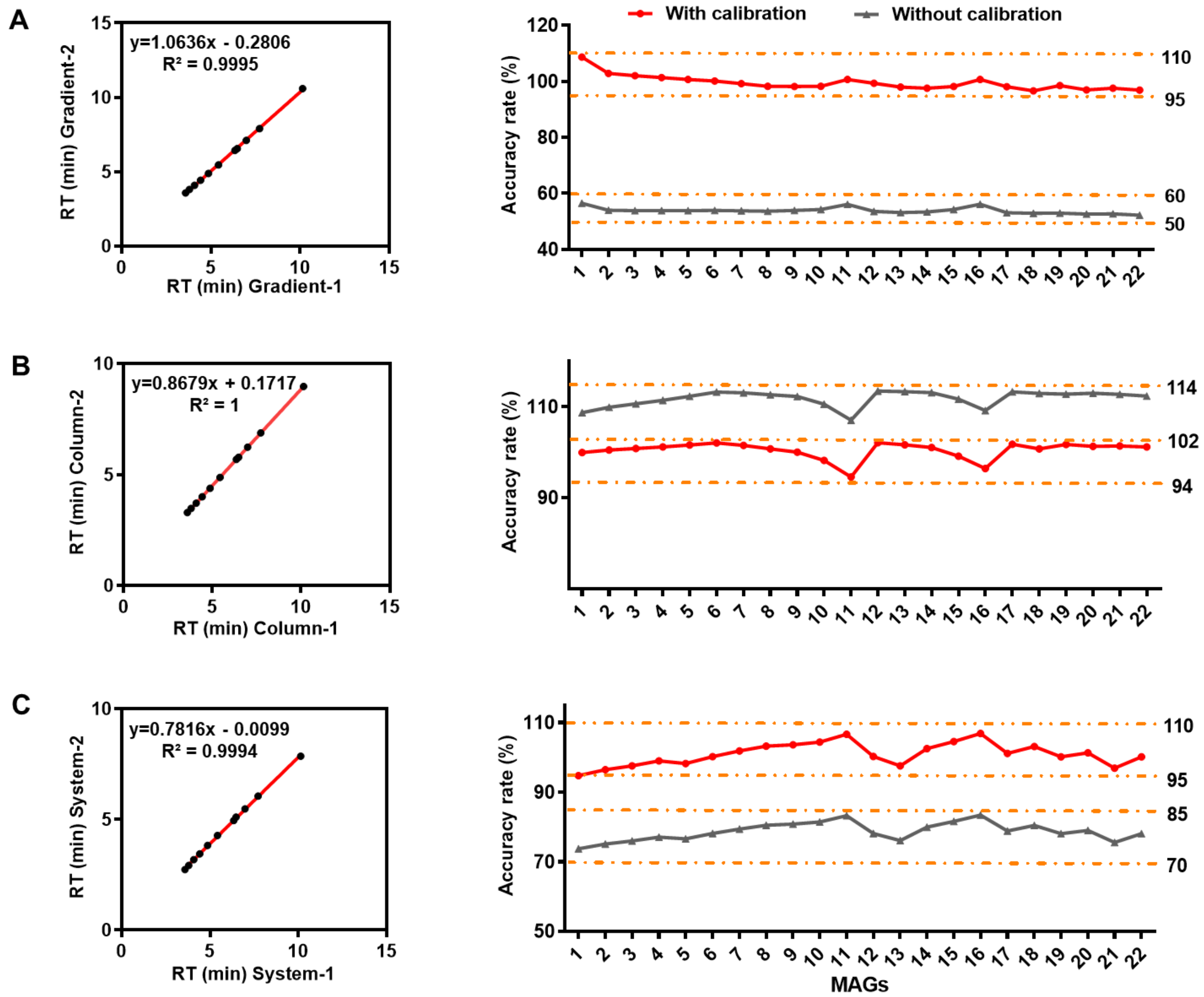

Figure S9. Regression analysis and calibration performance of retention times (RTs) for 1,2-PeD-MEs under different chromatographic conditions. (**A**) Gradient programs, (**B**) chromatographic columns, and (**C**) LC platforms. Corresponding RT prediction errors before (grey) and after (red) 1,2-PeD-ME-based correction are shown on the right. Detailed conditions are provided in **Table S4**.

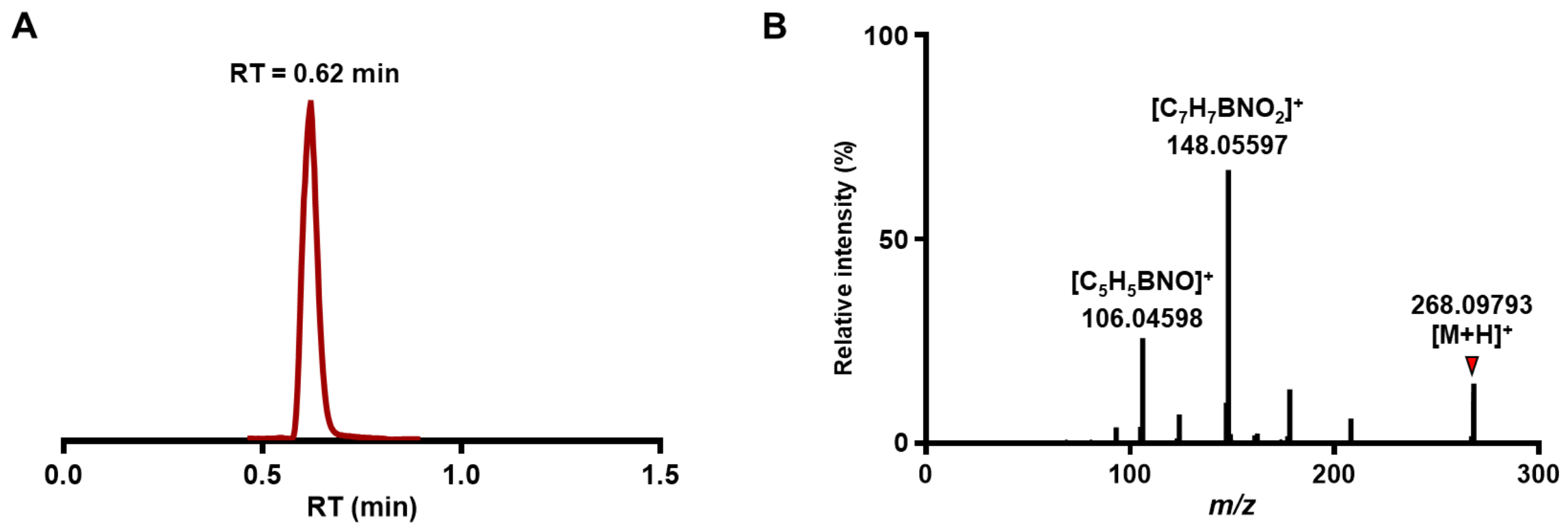

Figure S10. (**A**) Extracted ion chromatogram (EIC) and MS/MS spectrum of the 4-PyBA derivatized glucose.

Supporting Information

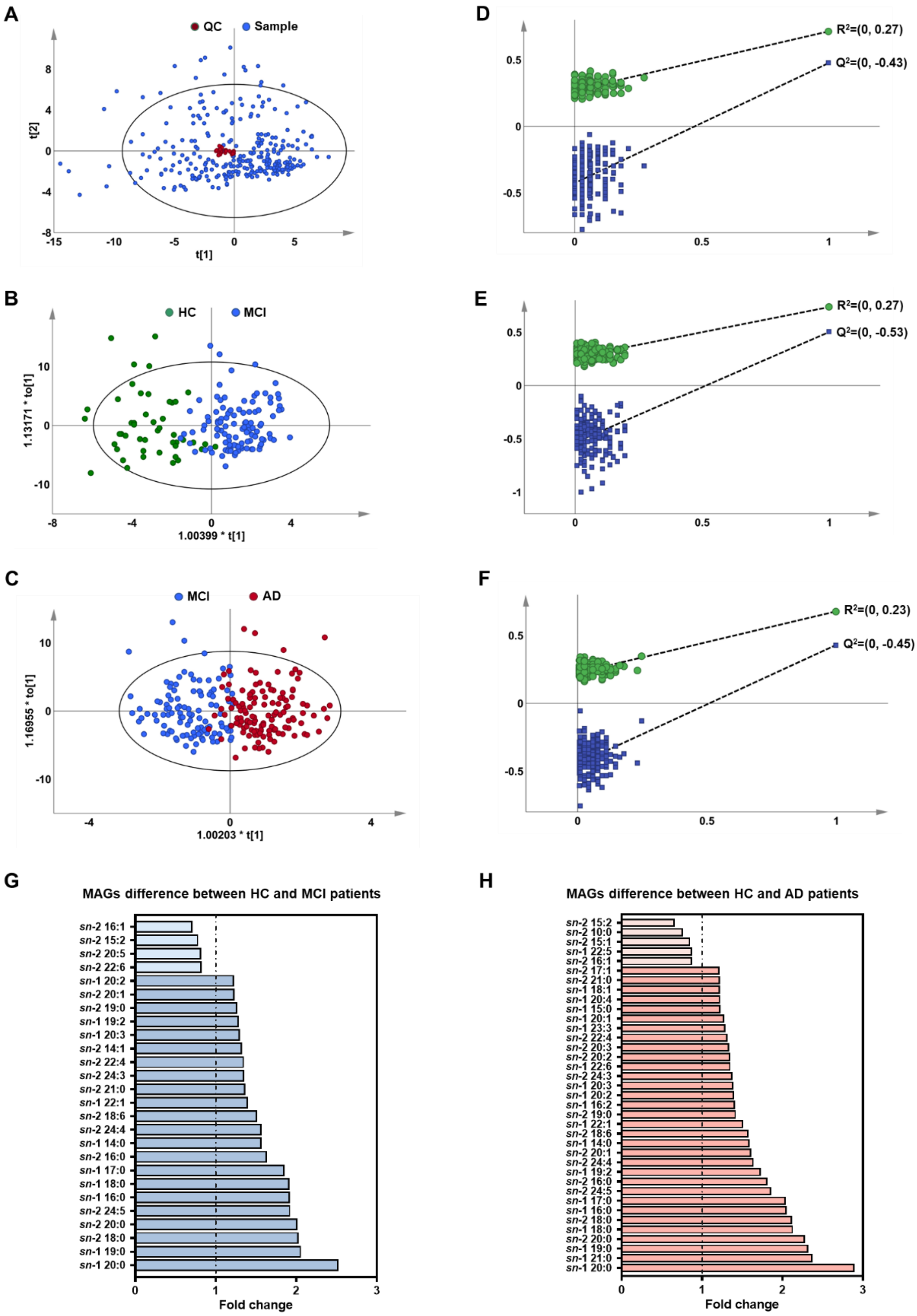

Figure S11. Serum MAG profiles in healthy controls (HC), mild cognitive impairment (MCI), and Alzheimer's disease (AD). (**A**) PCA score plot evaluating the analytical stability of the LC-MS platform. (**B**) Cross-validation tests comparing HC vs. AD. OPLS-DA score plots (left) and corresponding cross-validation tests (right) comparing (**C, D**) HC vs. MCI, and (**E, F**) MCI vs. AD. Differentially altered MAGs between (**G**) HC and MCI, and (**H**) HC and AD (fold change > 1.2 or < 0.8).

A

Analytes	PPARα	PPARβ	PPARγ	GPR119	TRPV1	CB1R	CB2R	GPR55
sn -1 MAG (14:0)	8.760	8.837	10.165	9.261	9.896	5.760	7.467	7.456
sn -1 MAG (16:0)	9.439	9.395	10.024	10.241	8.922	7.834	8.522	6.406
sn -1 MAG (17:0)	12.316	9.599	11.361	11.601	12.410	6.552	8.574	7.920
sn -1 MAG (18:0)	11.830	9.180	10.852	11.416	10.087	6.776	7.943	8.102
sn -1 MAG (20:0)	12.177	9.714	10.829	10.956	11.004	6.006	8.176	8.207
sn -2 MAG (20:3)	10.256	9.373	10.273	11.015	9.655	6.465	9.359	7.782

13

5

B

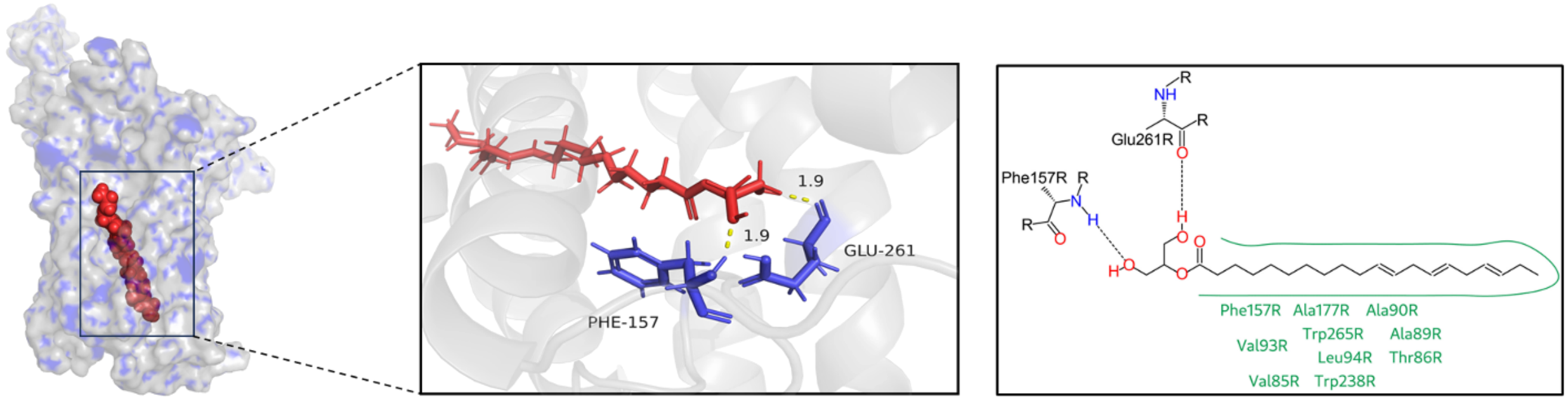

Figure S12. Molecular docking of six diagnostic MAG biomarkers with eight related receptors. (**A**) Heatmap of predicted binding affinities (docking scores), with color indicating binding strength (blue, low; red, high). (**B**) 3D binding conformation and 2D interaction map of sn-2 MAG (20:3) in the GPR119 pocket. Dashed lines indicate hydrogen bonds (Glu261 and Phe157; ~1.9 Å), and the green solid line denotes hydrophobic interactions.

Supplementary Tables

Table S1. Detailed information on 38 MAG regioisomer pairs (reference standards) and their corresponding 4-PyBA-labeled derivatives

No.	Abbreviation	RT (min)	Native MAGs Formula	Native MAGs Theoretical $[M+H]^+$	4-PyBA-labeled MAGs Formula	4-PyBA-labeled MAGs Theoretical $[M+H]^+$	Fold change[a]
1*	*sn*-1 MAG (6:0)	2.31	$C_9H_{18}O_4$	191.12779	$C_{14}H_{20}BNO_4$	278.15582	254
1'*	*sn*-2 MAG (6:0)	2.27	$C_9H_{18}O_4$	191.12779	$C_{14}H_{20}BNO_4$	278.15582	140
2*	*sn*-1 MAG (7:0)	2.44	$C_{10}H_{20}O_4$	205.14344	$C_{15}H_{22}BNO_4$	292.17147	131
2'*	*sn*-2 MAG (7:0)	2.42	$C_{10}H_{20}O_4$	205.14344	$C_{15}H_{22}BNO_4$	292.17147	212
3	*sn*-1 MAG (8:0)	2.58	$C_{11}H_{22}O_4$	219.15909	$C_{16}H_{24}BNO_4$	306.18712	105
3'	*sn*-2 MAG (8:0)	2.54	$C_{11}H_{22}O_4$	219.15909	$C_{16}H_{24}BNO_4$	306.18712	228
4	*sn*-1 MAG (10:0)	3.01	$C_{13}H_{26}O_4$	247.19039	$C_{18}H_{28}BNO_4$	334.21842	329
4'	*sn*-2 MAG (10:0)	2.92	$C_{13}H_{26}O_4$	247.19039	$C_{18}H_{28}BNO_4$	334.21842	366
5	*sn*-1 MAG (11:0)	3.34	$C_{14}H_{28}O_4$	261.20604	$C_{19}H_{30}BNO_4$	348.23407	180
5'	*sn*-2 MAG (11:0)	3.22	$C_{14}H_{28}O_4$	261.20604	$C_{19}H_{30}BNO_4$	348.23407	131
6	*sn*-1 MAG (12:0)	3.78	$C_{15}H_{30}O_4$	275.22169	$C_{20}H_{32}BNO_4$	362.24972	255
6'	*sn*-2 MAG (12:0)	3.63	$C_{15}H_{30}O_4$	275.22169	$C_{20}H_{32}BNO_4$	362.24972	72
7	*sn*-1 MAG (13:0)	4.39	$C_{16}H_{32}O_4$	289.23734	$C_{21}H_{34}BNO_4$	376.26537	230
7'	*sn*-2 MAG (13:0)	4.18	$C_{16}H_{32}O_4$	289.23734	$C_{21}H_{34}BNO_4$	376.26537	84
8	*sn*-1 MAG (14:0)	5.18	$C_{17}H_{34}O_4$	303.25299	$C_{22}H_{36}BNO_4$	390.28102	100
8'	*sn*-2 MAG (14:0)	4.91	$C_{17}H_{34}O_4$	303.25299	$C_{22}H_{36}BNO_4$	390.28102	295
9	*sn*-1 MAG (15:0)	6.18	$C_{18}H_{36}O_4$	317.26864	$C_{23}H_{38}BNO_4$	404.29667	120
9'	*sn*-2 MAG (15:0)	5.85	$C_{18}H_{36}O_4$	317.26864	$C_{23}H_{38}BNO_4$	404.29667	164
10	*sn*-1 MAG (16:0)	7.39	$C_{19}H_{38}O_4$	331.28429	$C_{24}H_{40}BNO_4$	418.31232	326
10'	*sn*-2 MAG (16:0)	6.97	$C_{19}H_{38}O_4$	331.28429	$C_{24}H_{40}BNO_4$	418.31232	78
11	*sn*-1 MAG (17:0)	8.78	$C_{20}H_{40}O_4$	345.29994	$C_{25}H_{42}BNO_4$	432.32797	161
11'	*sn*-2 MAG (17:0)	8.28	$C_{20}H_{40}O_4$	345.29994	$C_{25}H_{42}BNO_4$	432.32797	47
12	*sn*-1 MAG (18:0)	10.31	$C_{21}H_{42}O_4$	359.31559	$C_{26}H_{44}BNO_4$	446.34362	309
12'	*sn*-2 MAG (18:0)	9.76	$C_{21}H_{42}O_4$	359.31559	$C_{26}H_{44}BNO_4$	446.34362	61
13	*sn*-1 MAG (20:0)	13.48	$C_{23}H_{46}O_4$	387.34689	$C_{28}H_{48}BNO_4$	474.37492	142
13'	*sn*-2 MAG (20:0)	12.94	$C_{23}H_{46}O_4$	387.34689	$C_{28}H_{48}BNO_4$	474.37492	53
14*	*sn*-1 MAG (21:0)	15.09	$C_{24}H_{48}O_4$	401.36254	$C_{29}H_{50}BNO_4$	488.39057	76
14'*	*sn*-2 MAG (21:0)	14.48	$C_{24}H_{48}O_4$	401.36254	$C_{29}H_{50}BNO_4$	488.39057	27
15*	*sn*-1 MAG (22:0)	16.57	$C_{25}H_{50}O_4$	415.37819	$C_{30}H_{52}BNO_4$	502.40622	3
15'*	*sn*-2 MAG (22:0)	16.00	$C_{25}H_{50}O_4$	415.37819	$C_{30}H_{52}BNO_4$	502.40622	23
16*	*sn*-1 MAG (23:0)	17.99	$C_{26}H_{52}O_4$	429.39384	$C_{31}H_{54}BNO_4$	516.42187	8
16'*	*sn*-2 MAG (23:0)	17.41	$C_{26}H_{52}O_4$	429.39384	$C_{31}H_{54}BNO_4$	516.42187	71
17*	*sn*-1 MAG (24:0)	19.28	$C_{27}H_{54}O_4$	443.40949	$C_{32}H_{56}BNO_4$	530.43752	4
17'*	*sn*-2 MAG (24:0)	18.75	$C_{27}H_{54}O_4$	443.40949	$C_{32}H_{56}BNO_4$	530.43752	32
18*	*sn*-1 MAG (13:1)	4.02	$C_{16}H_{30}O_4$	287.22169	$C_{21}H_{32}BNO_4$	374.24972	18
18'*	*sn*-2 MAG (13:1)	3.86	$C_{16}H_{30}O_4$	287.22169	$C_{21}H_{32}BNO_4$	374.24972	27

19*	*sn*-1 MAG (14:1)	4.77	$C_{17}H_{32}O_4$	301.23734	$C_{22}H_{34}BNO_4$	388.26537	71
19'*	*sn*-2 MAG (14:1)	4.54	$C_{17}H_{32}O_4$	301.23734	$C_{22}H_{34}BNO_4$	388.26537	68
20	*sn*-1 MAG (16:1)	5.53	$C_{19}H_{36}O_4$	329.26864	$C_{24}H_{38}BNO_4$	416.29667	11
20'	*sn*-2 MAG (16:1)	5.24	$C_{19}H_{36}O_4$	329.26864	$C_{24}H_{38}BNO_4$	416.29667	24
21	*sn*-1 MAG (17:1)	6.52	$C_{20}H_{38}O_4$	343.28429	$C_{25}H_{40}BNO_4$	430.31232	55
21'	*sn*-2 MAG (17:1)	6.18	$C_{20}H_{38}O_4$	343.28429	$C_{25}H_{40}BNO_4$	430.31232	50
22	*sn*-1 MAG (18:1)	7.82	$C_{21}H_{40}O_4$	357.29994	$C_{26}H_{42}BNO_4$	444.32797	43
22'	*sn*-2 MAG (18:1)	7.41	$C_{21}H_{40}O_4$	357.29994	$C_{26}H_{42}BNO_4$	444.32797	45
23	*sn*-1 MAG (20:1)	10.61	$C_{23}H_{44}O_4$	385.33124	$C_{28}H_{46}BNO_4$	472.35927	67
23'	*sn*-2 MAG (20:1)	10.09	$C_{23}H_{44}O_4$	385.33124	$C_{28}H_{46}BNO_4$	472.35927	251
24	*sn*-1 MAG (22:1)	13.62	$C_{25}H_{48}O_4$	413.36254	$C_{30}H_{50}BNO_4$	500.39057	37
24'	*sn*-2 MAG (22:1)	13.07	$C_{25}H_{48}O_4$	413.36254	$C_{30}H_{50}BNO_4$	500.39057	51
25*	*sn*-1 MAG (24:1)	16.59	$C_{27}H_{52}O_4$	441.39384	$C_{32}H_{54}BNO_4$	528.42187	6
25'*	*sn*-2 MAG (24:1)	16.03	$C_{27}H_{52}O_4$	441.39384	$C_{32}H_{54}BNO_4$	528.42187	62
26	*sn*-1 MAG (18:2)	6.07	$C_{21}H_{38}O_4$	355.28429	$C_{26}H_{40}BNO_4$	442.31232	64
26'	*sn*-2 MAG (18:2)	5.75	$C_{21}H_{38}O_4$	355.28429	$C_{26}H_{40}BNO_4$	442.31232	24
27*	*sn*-1 MAG (19:2)	7.09	$C_{22}H_{40}O_4$	369.29994	$C_{27}H_{42}BNO_4$	456.32797	45
27'*	*sn*-2 MAG (19:2)	6.72	$C_{22}H_{40}O_4$	369.29994	$C_{27}H_{42}BNO_4$	456.32797	68
28	*sn*-1 MAG (20:2)	8.31	$C_{23}H_{42}O_4$	383.31559	$C_{28}H_{44}BNO_4$	470.34362	25
28'	*sn*-2 MAG (20:2)	7.89	$C_{23}H_{42}O_4$	383.31559	$C_{28}H_{44}BNO_4$	470.34362	37
29*	*sn*-1 MAG (21:2)	9.68	$C_{24}H_{44}O_4$	397.33124	$C_{29}H_{46}BNO_4$	484.35927	48
29'*	*sn*-2 MAG (21:2)	9.18	$C_{24}H_{44}O_4$	397.33124	$C_{29}H_{46}BNO_4$	484.35927	80
30*	*sn*-1 MAG (22:2)	11.11	$C_{25}H_{46}O_4$	411.34689	$C_{30}H_{48}BNO_4$	498.37492	68
30'*	*sn*-2 MAG (22:2)	10.60	$C_{25}H_{46}O_4$	411.34689	$C_{30}H_{48}BNO_4$	498.37492	118
31	*sn*-1 MAG (18:3)	5.09	$C_{21}H_{36}O_4$	353.26864	$C_{26}H_{38}BNO_4$	440.29667	28
31'	*sn*-2 MAG (18:3)	4.88	$C_{21}H_{36}O_4$	353.26864	$C_{26}H_{38}BNO_4$	440.29667	22
32	*sn*-1 MAG (20:3)	6.66	$C_{23}H_{40}O_4$	381.29994	$C_{28}H_{42}BNO_4$	468.32797	10
32'	*sn*-2 MAG (20:3)	6.33	$C_{23}H_{40}O_4$	381.29994	$C_{28}H_{42}BNO_4$	468.32797	27
33*	*sn*-1 MAG (22:3)	9.10	$C_{25}H_{44}O_4$	409.33124	$C_{30}H_{46}BNO_4$	496.35927	35
33'*	*sn*-2 MAG (22:3)	8.64	$C_{25}H_{44}O_4$	409.33124	$C_{30}H_{46}BNO_4$	496.35927	161
34*	*sn*-1 MAG (18:4)	4.21	$C_{21}H_{34}O_4$	351.25299	$C_{26}H_{36}BNO_4$	438.28102	33
34'*	*sn*-2 MAG (18:4)	4.08	$C_{21}H_{34}O_4$	351.25299	$C_{26}H_{36}BNO_4$	438.28102	20
35	*sn*-1 MAG (20:4)	5.82	$C_{23}H_{38}O_4$	379.28429	$C_{28}H_{40}BNO_4$	466.31232	14
35'	*sn*-2 MAG (20:4)	5.57	$C_{23}H_{38}O_4$	379.28429	$C_{28}H_{40}BNO_4$	466.31232	22
36*	*sn*-1 MAG (20:5)	4.77	$C_{23}H_{36}O_4$	377.26864	$C_{28}H_{38}BNO_4$	464.29667	15
36'*	*sn*-2 MAG (20:5)	4.61	$C_{23}H_{36}O_4$	377.26864	$C_{28}H_{38}BNO_4$	464.29667	42
37*	*sn*-1 MAG (22:5)	6.76	$C_{25}H_{40}O_4$	405.29994	$C_{30}H_{42}BNO_4$	492.32797	21
37'*	*sn*-2 MAG (22:5)	6.49	$C_{25}H_{40}O_4$	405.29994	$C_{30}H_{42}BNO_4$	492.32797	78
38	*sn*-1 MAG (22:6)	5.49	$C_{25}H_{38}O_4$	403.28429	$C_{30}H_{40}BNO_4$	490.31232	38
38'	*sn*-2 MAG (22:6)	5.30	$C_{25}H_{38}O_4$	403.28429	$C_{30}H_{40}BNO_4$	490.31232	33

* The synthesized MAGs.

[a] Fold change = peak area (4-PyBA-labeled MAG) / peak area (native MAG)

Supporting Information

Table S2. Detailed information on 16 fatty acid standards used for MAG synthesis

Fatty acids	Symbol	Chemical formula	MS (precursor ion)		MS/MS (product ion)	
			Formula	Theoretical $[M-H]^-$	Formula	Theoretical $[M-H-COO]^-$
Hexanoic acid	6:0	$C_6H_{12}O_2$	$C_6H_{11}O_2$	115.07645	C_5H_{11}	71.08662
Heptanoic acid	7:0	$C_7H_{14}O_2$	$C_7H_{13}O_2$	129.09210	C_6H_{13}	85.10227
Heneicosanoic acid	21:0	$C_{21}H_{42}O_2$	$C_{21}H_{41}O_2$	325.31120	$C_{20}H_{41}$	281.32137
Docosanoic acid	22:0	$C_{22}H_{44}O_2$	$C_{22}H_{43}O_2$	339.32685	$C_{21}H_{43}$	295.33702
Tricosanoic acid	23:0	$C_{23}H_{46}O_2$	$C_{23}H_{45}O_2$	353.34250	$C_{22}H_{45}$	309.35267
Lignoceric acid	24:0	$C_{24}H_{48}O_2$	$C_{24}H_{47}O_2$	367.35815	$C_{23}H_{47}$	323.36832
Tridecenoic acid	13:1 (n-11)	$C_{13}H_{24}O_2$	$C_{13}H_{23}O_2$	211.17035	$C_{12}H_{23}$	167.18052
Myristelaidic acid	14:1 (n-5)	$C_{14}H_{26}O_2$	$C_{14}H_{25}O_2$	225.18600	$C_{13}H_{25}$	181.19617
Nervonic acid	24:1 (n-9)	$C_{24}H_{46}O_2$	$C_{24}H_{45}O_2$	365.34250	$C_{23}H_{45}$	321.35267
Nonadecadienoic acid	19:2 (n-7)	$C_{19}H_{34}O_2$	$C_{19}H_{33}O_2$	293.24860	$C_{18}H_{33}$	249.25877
Heneicosadienoic acid	21:2 (n-5)	$C_{21}H_{38}O_2$	$C_{21}H_{37}O_2$	321.27990	$C_{20}H_{37}$	277.29007
Docosadienoic acid	22:2 (n-6)	$C_{22}H_{40}O_2$	$C_{22}H_{39}O_2$	335.29555	$C_{21}H_{39}$	291.30572
Docosatrienoic acid	22:3 (n-3)	$C_{22}H_{38}O_2$	$C_{22}H_{37}O_2$	333.27990	$C_{21}H_{37}$	289.29007
Stearidonic acid	18:4 (n-3)	$C_{18}H_{28}O_2$	$C_{18}H_{27}O_2$	275.20165	$C_{17}H_{27}$	231.21182
Eicosapentaenoic acid	20:5 (n-3)	$C_{20}H_{30}O_2$	$C_{20}H_{29}O_2$	301.21730	$C_{19}H_{29}$	257.22747
Docosapentaenoic acid	22:5 (n-3)	$C_{22}H_{34}O_2$	$C_{22}H_{33}O_2$	329.24860	$C_{21}H_{33}$	285.25877

Table S3. Ten 1,2-PeD-MEs used for the RT calibration

No.	Analytes	Formula	Theoretical MS value	
			MS *m/z* $[M+H]^+$	MS/MS *m/z* $[C_5H_9]^+$
1”	1,2-PeD-ME (6:0)	$C_{11}H_{22}O_3$	203.16417	69.06988
2”	1,2-PeD-ME (7:0)	$C_{12}H_{24}O_3$	217.17982	69.06988
3”	1,2-PeD-ME (8:0)	$C_{13}H_{26}O_3$	231.19547	69.06988
4”	1,2-PeD-ME (9:0)	$C_{14}H_{28}O_3$	245.21112	69.06988
5”	1,2-PeD-ME (10:0)	$C_{15}H_{30}O_3$	259.22677	69.06988
6”	1,2-PeD-ME (11:0)	$C_{16}H_{32}O_3$	273.24242	69.06988
7”	1,2-PeD-ME (13:1)	$C_{18}H_{34}O_3$	299.25807	69.06988
8”	1,2-PeD-ME (20:5)	$C_{25}H_{40}O_3$	389.30502	69.06988
9”	1,2-PeD-ME (22:6)	$C_{27}H_{42}O_3$	415.32067	69.06988
10”	1,2-PeD-ME (22:4)	$C_{27}H_{46}O_3$	419.35197	69.06988

Table S4. LC gradient programs, columns, and instrument systems used in RT calibration experiments

**(A) LC gradients (A: water; B: ACN)**			
Gradient 1		Gradient 2	
time (min)	B (%)	time (min)	B (%)
0-0.5	10	0-0.5	10
0.5-1	10-80	0.5-1	10-80
1-15	80-100	1-20	80-100
15-17	100-100	20-21	100-100
**(B) Columns**			
Column 1	Waters ACQUITY UPLC BEH C_{18} (100 mm × 2.1 mm, 1.7 µm)		
Column 2	SHIM-PACK Velox C_{18} (100 mm × 2.1 mm, 1.8 µm)		
**(C) UHPLC systems**			
System 1	Vanquish™ Flex UHPLC (Thermo Fisher Scientific, USA)		
System 2	UltiMate™ 3000 UHPLC (Thermo Fisher Scientific, USA)		

Table S5. Detailed information on the in-house database of *sn*-1 and *sn*-2 MAGs

No.	MAGs	Formula	Theoretical predicted value (*m/z*)			RT (min)	
			MS	$[C_5H_5ONB]^+$	$[C_8H_9O_2NB]^+$	*sn*-1	*sn*-2
D1	MAG (6:0)	$C_{14}H_{20}BNO_4$	278.15582	106.04587	162.07209	2.31	2.27
D2	MAG (7:0)	$C_{15}H_{22}BNO_4$	292.17147	106.04587	162.07209	2.44	2.42
D3	MAG (8:0)	$C_{16}H_{24}BNO_4$	306.18712	106.04587	162.07209	2.58	2.54
D4	MAG (9:0)	$C_{17}H_{26}BNO_4$	320.20277	106.04587	162.07209	2.89	2.80
D5	MAG (10:0)	$C_{18}H_{28}BNO_4$	334.21842	106.04587	162.07209	3.01	2.92
D6	MAG (11:0)	$C_{19}H_{30}BNO_4$	348.23407	106.04587	162.07209	3.34	3.22
D7	MAG (12:0)	$C_{20}H_{32}BNO_4$	362.24972	106.04587	162.07209	3.78	3.63
D8	MAG (13:0)	$C_{21}H_{34}BNO_4$	376.26537	106.04587	162.07209	4.39	4.18
D9	MAG (14:0)	$C_{22}H_{36}BNO_4$	390.28102	106.04587	162.07209	5.18	4.91
D10	MAG (15:0)	$C_{23}H_{38}BNO_4$	404.29667	106.04587	162.07209	6.18	5.85
D11	MAG (16:0)	$C_{24}H_{40}BNO_4$	418.31232	106.04587	162.07209	7.39	6.97
D12	MAG (17:0)	$C_{25}H_{42}BNO_4$	432.32797	106.04587	162.07209	8.78	8.28
D13	MAG (18:0)	$C_{26}H_{44}BNO_4$	446.34362	106.04587	162.07209	10.31	9.76
D14	MAG (19:0)	$C_{27}H_{46}BNO_4$	460.35927	106.04587	162.07209	10.78	10.33
D15	MAG (20:0)	$C_{28}H_{48}BNO_4$	474.37492	106.04587	162.07209	13.48	12.94
D16	MAG (21:0)	$C_{29}H_{50}BNO_4$	488.39057	106.04587	162.07209	15.09	14.48
D17	MAG (22:0)	$C_{30}H_{52}BNO_4$	502.40622	106.04587	162.07209	16.57	16.00
D18	MAG (23:0)	$C_{31}H_{54}BNO_4$	516.42187	106.04587	162.07209	17.99	17.41
D19	MAG (24:0)	$C_{32}H_{56}BNO_4$	530.43752	106.04587	162.07209	19.28	18.75
D20	MAG (25:0)	$C_{33}H_{58}BNO_4$	544.45317	106.04587	162.07209	23.73	22.59
D21	MAG (6:1)	$C_{14}H_{18}BNO_4$	276.14017	106.04587	162.07209	1.58	1.45
D22	MAG (7:1)	$C_{15}H_{20}BNO_4$	290.15582	106.04587	162.07209	1.80	1.66
D23	MAG (8:1)	$C_{16}H_{22}BNO_4$	304.17147	106.04587	162.07209	2.06	1.90
D24	MAG (9:1)	$C_{17}H_{24}BNO_4$	318.18712	106.04587	162.07209	2.35	2.18
D25	MAG (10:1)	$C_{18}H_{26}BNO_4$	332.20277	106.04587	162.07209	2.68	2.50
D26	MAG (11:1)	$C_{19}H_{28}BNO_4$	346.21842	106.04587	162.07209	3.06	2.86
D27	MAG (12:1)	$C_{20}H_{30}BNO_4$	360.23407	106.04587	162.07209	3.50	3.28
D28	MAG (13:1)	$C_{21}H_{32}BNO_4$	374.24972	106.04587	162.07209	4.02	3.86
D29	MAG (14:1)	$C_{22}H_{34}BNO_4$	388.26537	106.04587	162.07209	4.77	4.54
D30	MAG (15:1)	$C_{23}H_{36}BNO_4$	402.28102	106.04587	162.07209	5.21	4.94
D31	MAG (16:1)	$C_{24}H_{38}BNO_4$	416.29667	106.04587	162.07209	5.53	5.24
D32	MAG (17:1)	$C_{25}H_{40}BNO_4$	430.31232	106.04587	162.07209	6.52	6.18
D33	MAG (18:1)	$C_{26}H_{42}BNO_4$	444.32797	106.04587	162.07209	7.82	7.41
D34	MAG (19:1)	$C_{27}H_{44}BNO_4$	458.34362	106.04587	162.07209	8.85	8.52
D35	MAG (20:1)	$C_{28}H_{46}BNO_4$	472.35927	106.04587	162.07209	10.61	10.09
D36	MAG (21:1)	$C_{29}H_{48}BNO_4$	486.37492	106.04587	162.07209	11.54	11.19
D37	MAG (22:1)	$C_{30}H_{50}BNO_4$	500.39057	106.04587	162.07209	13.62	13.07
D38	MAG (23:1)	$C_{31}H_{52}BNO_4$	514.40622	106.04587	162.07209	15.04	14.70
D39	MAG (24:1)	$C_{32}H_{54}BNO_4$	528.42187	106.04587	162.07209	16.59	16.03
D40	MAG (25:1)	$C_{33}H_{56}BNO_4$	542.43752	106.04587	162.07209	19.61	19.32
D41	MAG (26:1)	$C_{34}H_{58}BNO_4$	556.45317	106.04587	162.07209	22.39	22.14
D42	MAG (27:1)	$C_{35}H_{60}BNO_4$	570.46882	106.04587	162.07209	25.57	25.37
D43	MAG (6:2)	$C_{14}H_{16}BNO_4$	274.12452	106.04587	162.07209	0.98	0.91
D44	MAG (7:2)	$C_{15}H_{18}BNO_4$	288.14017	106.04587	162.07209	1.14	1.07
D45	MAG (8:2)	$C_{16}H_{20}BNO_4$	302.15582	106.04587	162.07209	1.33	1.24
D46	MAG (9:2)	$C_{17}H_{22}BNO_4$	316.17147	106.04587	162.07209	1.55	1.45
D47	MAG (10:2)	$C_{18}H_{24}BNO_4$	330.18712	106.04587	162.07209	1.81	1.69
D48	MAG (11:2)	$C_{19}H_{26}BNO_4$	344.20277	106.04587	162.07209	2.10	1.97
D49	MAG (12:2)	$C_{20}H_{28}BNO_4$	358.21842	106.04587	162.07209	2.45	2.30
D50	MAG (13:2)	$C_{21}H_{30}BNO_4$	372.23407	106.04587	162.07209	2.85	2.68
D51	MAG (14:2)	$C_{22}H_{32}BNO_4$	386.24972	106.04587	162.07209	3.32	3.12

D52	MAG (15:2)	$C_{23}H_{34}BNO_4$	400.26537	106.04587	162.07209	3.86	3.64
D53	MAG (16:2)	$C_{24}H_{36}BNO_4$	414.28102	106.04587	162.07209	4.49	4.24
D54	MAG (17:2)	$C_{25}H_{38}BNO_4$	428.29667	106.04587	162.07209	5.23	4.95
D55	MAG (18:2)	$C_{26}H_{40}BNO_4$	442.31232	106.04587	162.07209	6.07	5.75
D56	MAG (19:2)	$C_{27}H_{42}BNO_4$	456.32797	106.04587	162.07209	7.09	6.72
D57	MAG (20:2)	$C_{28}H_{44}BNO_4$	470.34362	106.04587	162.07209	8.31	7.89
D58	MAG (21:2)	$C_{29}H_{46}BNO_4$	484.35927	106.04587	162.07209	9.68	9.18
D59	MAG (22:2)	$C_{30}H_{48}BNO_4$	498.37492	106.04587	162.07209	11.11	10.60
D60	MAG (23:2)	$C_{31}H_{50}BNO_4$	512.39057	106.04587	162.07209	13.03	12.42
D61	MAG (24:2)	$C_{32}H_{52}BNO_4$	526.40622	106.04587	162.07209	15.16	14.49
D62	MAG (25:2)	$C_{33}H_{54}BNO_4$	540.42187	106.04587	162.07209	17.65	16.89
D63	MAG (26:2)	$C_{34}H_{56}BNO_4$	554.43752	106.04587	162.07209	20.55	19.69
D64	MAG (27:2)	$C_{35}H_{58}BNO_4$	568.45317	106.04587	162.07209	23.92	22.96
D65	MAG (6:3)	$C_{14}H_{14}BNO_4$	272.10886	106.04587	162.07209	0.88	0.87
D66	MAG (7:3)	$C_{15}H_{16}BNO_4$	286.12452	106.04587	162.07209	1.02	1.01
D67	MAG (8:3)	$C_{16}H_{18}BNO_4$	300.14017	106.04587	162.07209	1.18	1.16
D68	MAG (9:3)	$C_{17}H_{20}BNO_4$	314.15582	106.04587	162.07209	1.37	1.34
D69	MAG (10:3)	$C_{18}H_{22}BNO_4$	328.17147	106.04587	162.07209	1.58	1.54
D70	MAG (11:3)	$C_{19}H_{24}BNO_4$	342.18712	106.04587	162.07209	1.83	1.78
D71	MAG (12:3)	$C_{20}H_{26}BNO_4$	356.20277	106.04587	162.07209	2.11	2.05
D72	MAG (13:3)	$C_{21}H_{28}BNO_4$	370.21842	106.04587	162.07209	2.44	2.37
D73	MAG (14:3)	$C_{22}H_{30}BNO_4$	384.23407	106.04587	162.07209	2.82	2.73
D74	MAG (15:3)	$C_{23}H_{32}BNO_4$	398.24972	106.04587	162.07209	3.27	3.15
D75	MAG (16:3)	$C_{24}H_{34}BNO_4$	412.26537	106.04587	162.07209	3.78	3.64
D76	MAG (17:3)	$C_{25}H_{36}BNO_4$	426.28102	106.04587	162.07209	4.37	4.19
D77	MAG (18:3)	$C_{26}H_{38}BNO_4$	440.29667	106.04587	162.07209	5.09	4.88
D78	MAG (19:3)	$C_{27}H_{40}BNO_4$	454.31232	106.04587	162.07209	5.84	5.58
D79	MAG (20:3)	$C_{28}H_{42}BNO_4$	468.32797	106.04587	162.07209	6.66	6.33
D80	MAG (21:3)	$C_{29}H_{44}BNO_4$	482.34362	106.04587	162.07209	7.80	7.42
D81	MAG (22:3)	$C_{30}H_{46}BNO_4$	496.35927	106.04587	162.07209	9.10	8.64
D82	MAG (23:3)	$C_{31}H_{48}BNO_4$	510.37492	106.04587	162.07209	10.43	9.88
D83	MAG (24:3)	$C_{32}H_{50}BNO_4$	524.39057	106.04587	162.07209	12.06	11.40
D84	MAG (25:3)	$C_{33}H_{52}BNO_4$	538.40622	106.04587	162.07209	13.95	13.14
D85	MAG (26:3)	$C_{34}H_{54}BNO_4$	552.42187	106.04587	162.07209	16.13	15.16
D86	MAG (27:3)	$C_{35}H_{56}BNO_4$	566.43752	106.04587	162.07209	18.65	17.49
D87	MAG (28:3)	$C_{36}H_{58}BNO_4$	580.45317	106.04587	162.07209	21.57	20.17
D88	MAG (29:3)	$C_{37}H_{60}BNO_4$	594.46882	106.04587	162.07209	24.94	23.27
D89	MAG (10:4)	$C_{18}H_{20}BNO_4$	326.15582	106.04587	162.07209	1.33	1.32
D90	MAG (11:4)	$C_{19}H_{22}BNO_4$	340.17147	106.04587	162.07209	1.54	1.52
D91	MAG (12:4)	$C_{20}H_{24}BNO_4$	354.18712	106.04587	162.07209	1.78	1.76
D92	MAG (13:4)	$C_{21}H_{26}BNO_4$	368.20277	106.04587	162.07209	2.06	2.02
D93	MAG (14:4)	$C_{22}H_{28}BNO_4$	382.21842	106.04587	162.07209	2.38	2.33
D94	MAG (15:4)	$C_{23}H_{30}BNO_4$	396.23407	106.04587	162.07209	2.75	2.69
D95	MAG (16:4)	$C_{24}H_{32}BNO_4$	410.24972	106.04587	162.07209	3.18	3.10
D96	MAG (17:4)	$C_{25}H_{34}BNO_4$	424.26537	106.04587	162.07209	3.68	3.57
D97	MAG (18:4)	$C_{26}H_{36}BNO_4$	438.28102	106.04587	162.07209	4.21	4.08
D98	MAG (19:4)	$C_{27}H_{38}BNO_4$	452.29667	106.04587	162.07209	4.92	4.75
D99	MAG (20:4)	$C_{28}H_{40}BNO_4$	466.31232	106.04587	162.07209	5.82	5.57
D100	MAG (21:4)	$C_{29}H_{42}BNO_4$	480.32797	106.04587	162.07209	6.57	6.30
D101	MAG (22:4)	$C_{30}H_{44}BNO_4$	494.34362	106.04587	162.07209	7.52	7.20
D102	MAG (23:4)	$C_{31}H_{46}BNO_4$	508.35927	106.04587	162.07209	8.79	8.38
D103	MAG (24:4)	$C_{32}H_{48}BNO_4$	522.37492	106.04587	162.07209	10.16	9.65
D104	MAG (25:4)	$C_{33}H_{50}BNO_4$	536.39057	106.04587	162.07209	11.74	11.13
D105	MAG (26:4)	$C_{34}H_{52}BNO_4$	550.40622	106.04587	162.07209	13.57	12.82
D106	MAG (27:4)	$C_{35}H_{54}BNO_4$	564.42187	106.04587	162.07209	15.69	14.78
D107	MAG (28:4)	$C_{36}H_{56}BNO_4$	578.43752	106.04587	162.07209	18.14	17.04
D108	MAG (29:4)	$C_{37}H_{58}BNO_4$	592.45317	106.04587	162.07209	20.97	19.64

D109	MAG (30:4)	$C_{38}H_{60}BNO_4$	606.46882	106.04587	162.07209	24.24	22.63
D110	MAG (12:5)	$C_{20}H_{22}BNO_4$	352.17147	106.04587	162.07209	1.11	1.10
D111	MAG (13:5)	$C_{21}H_{24}BNO_4$	366.18712	106.04587	162.07209	1.33	1.31
D112	MAG (14:5)	$C_{22}H_{26}BNO_4$	380.20277	106.04587	162.07209	1.59	1.57
D113	MAG (15:5)	$C_{23}H_{28}BNO_4$	394.21842	106.04587	162.07209	1.91	1.87
D114	MAG (16:5)	$C_{24}H_{30}BNO_4$	408.23407	106.04587	162.07209	2.29	2.24
D115	MAG (17:5)	$C_{25}H_{32}BNO_4$	422.24972	106.04587	162.07209	2.74	2.67
D116	MAG (18:5)	$C_{26}H_{34}BNO_4$	436.26537	106.04587	162.07209	3.27	3.18
D117	MAG (19:5)	$C_{27}H_{36}BNO_4$	450.28102	106.04587	162.07209	3.94	3.82
D118	MAG (20:5)	$C_{28}H_{38}BNO_4$	464.29667	106.04587	162.07209	4.77	4.61
D119	MAG (21:5)	$C_{29}H_{40}BNO_4$	478.31232	106.04587	162.07209	5.66	5.46
D120	MAG (22:5)	$C_{30}H_{42}BNO_4$	492.32797	106.04587	162.07209	6.76	6.49
D121	MAG (23:5)	$C_{31}H_{44}BNO_4$	506.34362	106.04587	162.07209	8.13	7.80
D122	MAG (24:5)	$C_{32}H_{46}BNO_4$	520.35927	106.04587	162.07209	9.75	9.32
D123	MAG (25:5)	$C_{33}H_{48}BNO_4$	534.37492	106.04587	162.07209	11.69	11.14
D124	MAG (26:5)	$C_{34}H_{50}BNO_4$	548.39057	106.04587	162.07209	14.01	13.32
D125	MAG (27:5)	$C_{35}H_{52}BNO_4$	562.40622	106.04587	162.07209	16.80	15.92
D126	MAG (28:5)	$C_{36}H_{54}BNO_4$	576.42187	106.04587	162.07209	20.14	19.02
D127	MAG (29:5)	$C_{37}H_{56}BNO_4$	590.43752	106.04587	162.07209	24.14	22.74
D128	MAG (14:6)	$C_{22}H_{24}BNO_4$	378.18712	106.04587	162.07209	1.25	1.23
D129	MAG (15:6)	$C_{23}H_{26}BNO_4$	392.20277	106.04587	162.07209	1.50	1.48
D130	MAG (16:6)	$C_{24}H_{28}BNO_4$	406.21842	106.04587	162.07209	1.81	1.77
D131	MAG (17:6)	$C_{25}H_{30}BNO_4$	420.23407	106.04587	162.07209	2.18	2.12
D132	MAG (18:6)	$C_{26}H_{32}BNO_4$	434.24972	106.04587	162.07209	2.62	2.56
D133	MAG (19:6)	$C_{27}H_{34}BNO_4$	448.26537	106.04587	162.07209	3.15	3.05
D134	MAG (20:6)	$C_{28}H_{36}BNO_4$	462.28102	106.04587	162.07209	3.78	3.62
D135	MAG (21:6)	$C_{29}H_{38}BNO_4$	476.29667	106.04587	162.07209	4.56	4.39
D136	MAG (22:6)	$C_{30}H_{40}BNO_4$	490.31232	106.04587	162.07209	5.49	5.30
D137	MAG (23:6)	$C_{31}H_{42}BNO_4$	504.32797	106.04587	162.07209	6.59	6.32
D138	MAG (24:6)	$C_{32}H_{44}BNO_4$	518.34362	106.04587	162.07209	7.93	7.57
D139	MAG (25:6)	$C_{33}H_{46}BNO_4$	532.35927	106.04587	162.07209	9.54	9.08
D140	MAG (26:6)	$C_{34}H_{48}BNO_4$	546.37492	106.04587	162.07209	11.48	10.89
D141	MAG (27:6)	$C_{35}H_{50}BNO_4$	560.39057	106.04587	162.07209	13.81	13.06
D142	MAG (28:6)	$C_{36}H_{52}BNO_4$	574.40622	106.04587	162.07209	16.61	15.67
D143	MAG (29:6)	$C_{37}H_{54}BNO_4$	588.42187	106.04587	162.07209	19.98	18.79
D144	MAG (30:6)	$C_{38}H_{56}BNO_4$	602.43752	106.04587	162.07209	24.04	22.53

Table S6. Detailed information on 118 *sn*-1 and *sn*-2 MAGs identified in biological samples

Analytes	Formula	Theoretical predicted value			Experimental value (QC)			ppm		RT accuracy (%)	Database	
		MS	MS/MS	RT (min)	MS	MS/MS	RT (min)	MS	MS/MS		LIPID MAPS	HMDB
sn-1 MAG (13:0) [a]	$C_{21}H_{34}BNO_4$	376.26537	106.04587	4.90	376.26337	106.04618	4.35	-5.304	2.915	88.78		√
sn-1 MAG (14:0) [a]	$C_{22}H_{36}BNO_4$	390.28102	106.04587	5.59	390.28061	106.04617	5.13	-1.039	2.821	91.69		√
sn-1 MAG (15:0) [a]	$C_{23}H_{38}BNO_4$	404.29667	106.04587	6.39	404.29584	106.04617	6.13	-2.042	2.821	95.95	√	√
sn-1 MAG (16:0) [a]	$C_{24}H_{40}BNO_4$	418.31232	106.04587	7.30	418.31232	106.04618	7.24	0.010	2.915	99.24	√	√
sn-1 MAG (17:0) [a]	$C_{25}H_{42}BNO_4$	432.32797	106.04587	8.33	432.32788	106.04614	8.70	-0.198	2.538	104.43	√	√
sn-1 MAG (18:0) [a]	$C_{26}H_{44}BNO_4$	446.31232	106.04587	9.51	446.34344	106.04616	10.21	-0.394	2.727	107.33	√	√
sn-1 MAG (19:0) [b]	$C_{27}H_{46}BNO_4$	460.35927	106.04587	10.86	460.35898	106.04617	11.79	-0.621	2.821	108.53		√
sn-1 MAG (20:0) [a]	$C_{28}H_{48}BNO_4$	474.37492	106.04587	12.40	474.37393	106.04610	13.37	-2.078	2.161	107.78	√	√
sn-1 MAG (21:0) [a]	$C_{29}H_{50}BNO_4$	488.39057	106.04587	14.16	488.38959	106.04618	14.85	-1.998	2.915	104.84		√
sn-1 MAG (22:0) [a]	$C_{30}H_{52}BNO_4$	502.40622	106.04587	16.17	502.40588	106.04618	16.34	-0.669	2.915	101.02		√
sn-1 MAG (23:0) [a]	$C_{31}H_{54}BNO_4$	516.42187	106.04587	18.47	516.42706	106.04616	17.78	10.057	2.727	96.26		
sn-1 MAG (24:0) [a]	$C_{32}H_{56}BNO_4$	530.43752	106.04587	21.09	530.43811	106.04622	19.10	1.119	3.292	90.56		√
sn-1 MAG (25:0) [b]	$C_{33}H_{58}BNO_4$	544.45317	106.04587	24.08	544.46484	106.04627	20.29	21.441	3.764	84.25		√
sn-2 MAG (8:0) [a]	$C_{16}H_{24}BNO_4$	306.18712	106.04587	2.45	306.18610	106.04617	2.59	-3.316	2.821	105.74		
sn-2 MAG (9:0) [b]	$C_{17}H_{26}BNO_4$	320.20277	106.04587	2.79	320.20114	106.04616	2.72	-5.076	2.727	97.38		
sn-2 MAG (10:0) [a]	$C_{18}H_{28}BNO_4$	334.21842	106.04587	3.19	334.21750	106.04604	2.91	-2.739	1.595	91.35		√
sn-2 MAG (11:0) [a]	$C_{19}H_{30}BNO_4$	348.23407	106.04587	3.63	348.23349	106.04618	3.32	-1.652	2.915	91.39		
sn-2 MAG (12:0) [a]	$C_{20}H_{32}BNO_4$	362.24972	106.04587	4.14	362.25177	106.04622	3.78	5.671	3.292	91.24		√
sn-2 MAG (13:0) [a]	$C_{21}H_{34}BNO_4$	376.26537	106.04587	4.72	376.26489	106.04620	4.16	-1.264	3.104	88.05		√
sn-2 MAG (14:0) [a]	$C_{22}H_{36}BNO_4$	390.28102	106.04587	5.39	390.28091	106.04617	4.87	-0.271	2.821	90.38		√
sn-2 MAG (15:0) [a]	$C_{23}H_{38}BNO_4$	404.29667	106.04587	6.14	404.29626	106.04613	5.80	-1.003	2.444	94.39		√

sn-2 MAG (16:0) [a]	$C_{24}H_{40}BNO_4$	418.31232	106.04587	7.01	418.31216	106.04618	6.88	-0.372	2.915	98.18	√	√
sn-2 MAG (17:0) [a]	$C_{25}H_{42}BNO_4$	432.32797	106.04587	7.99	432.32794	106.04617	8.21	-0.060	2.821	102.73		√
sn-2 MAG (18:0) [a]	$C_{26}H_{44}BNO_4$	446.31232	106.04587	9.11	446.34409	106.04615	9.67	1.062	2.632	106.10	√	√
sn-2 MAG (19:0) [b]	$C_{27}H_{46}BNO_4$	460.35927	106.04587	10.39	460.35858	106.04617	11.20	-1.490	2.821	107.75		√
sn-2 MAG (20:0) [a]	$C_{28}H_{48}BNO_4$	474.37492	106.04587	11.85	474.37515	106.04621	12.75	0.493	3.198	107.56		√
sn-2 MAG (21:0) [a]	$C_{29}H_{50}BNO_4$	488.39057	106.04587	13.52	488.39023	106.04620	14.29	-0.688	3.104	105.71		√
sn-2 MAG (22:0) [a]	$C_{30}H_{52}BNO_4$	502.40622	106.04587	15.42	502.40637	106.04618	15.79	0.306	2.915	102.42		√
sn-2 MAG (23:0) [a]	$C_{31}H_{54}BNO_4$	516.42187	106.04587	17.58	516.42194	106.04620	17.19	0.143	3.104	97.77		
sn-2 MAG (24:0) [a]	$C_{32}H_{56}BNO_4$	530.43752	106.04587	20.05	530.43774	106.04618	18.55	0.422	2.915	92.52		√
sn-2 MAG (25:0) [b]	$C_{33}H_{58}BNO_4$	544.45317	106.04587	22.87	544.45026	106.04615	19.76	-5.338	2.632	86.42		√
sn-1 MAG (14:1) [a]	$C_{22}H_{34}BNO_4$	388.26537	106.04587	4.56	/	106.04619	4.80	/	3.010	105.25		√
sn-1 MAG (15:1) [b]	$C_{23}H_{36}BNO_4$	402.28102	106.04587	5.21	402.27975	106.04619	4.91	-3.146	3.010	94.29		
sn-1 MAG (16:1) [a]	$C_{24}H_{38}BNO_4$	416.29667	106.04587	5.95	416.29727	106.04618	5.48	1.452	2.915	92.17		
sn-1 MAG (17:1) [a]	$C_{25}H_{40}BNO_4$	430.31232	106.04587	6.79	430.31207	106.04618	6.50	-0.571	2.915	95.75		
sn-1 MAG (18:1) [a]	$C_{26}H_{42}BNO_4$	444.32797	106.04587	7.75	444.32794	106.04617	7.74	-0.058	2.821	99.86	√	√
sn-1 MAG (19:1) [b]	$C_{27}H_{44}BNO_4$	458.34362	106.04587	8.85	458.34335	106.04619	8.90	-0.580	3.010	100.56		
sn-1 MAG (20:1) [a]	$C_{28}H_{46}BNO_4$	472.35927	106.04587	10.10	472.35950	106.04618	10.48	0.496	2.915	103.71		
sn-1 MAG (21:1) [b]	$C_{29}H_{48}BNO_4$	486.37492	106.04587	11.54	486.37170	106.04618	11.91	-6.612	2.915	103.23		
sn-1 MAG (22:1) [a]	$C_{30}H_{50}BNO_4$	500.39057	106.04587	13.17	500.39157	106.04621	13.49	2.006	3.198	102.40		√
sn-1 MAG (23:1) [b]	$C_{31}H_{52}BNO_4$	514.40622	106.04587	15.04	514.40747	106.04623	14.98	2.438	3.387	99.59		
sn-1 MAG (24:1) [a]	$C_{32}H_{54}BNO_4$	528.42187	106.04587	17.17	528.42438	106.04621	16.42	4.757	3.198	95.61		√
sn-2 MAG (12:1) [b]	$C_{20}H_{30}BNO_4$	360.23407	106.04587	3.28	/	106.04613	3.25	/	2.444	99.10		
sn-2 MAG (14:1) [a]	$C_{22}H_{34}BNO_4$	388.26537	106.04587	4.31	/	106.04620	4.74	/	3.104	110.03		√
sn-2 MAG (15:1) [b]	$C_{23}H_{36}BNO_4$	402.28102	106.04587	4.94	402.28113	106.04619	4.73	0.284	3.010	95.79		
sn-2 MAG (16:1) [a]	$C_{24}H_{38}BNO_4$	416.29667	106.04587	5.66	416.29669	106.04617	5.20	0.058	2.821	91.88		√
sn-2 MAG (17:1) [a]	$C_{25}H_{40}BNO_4$	430.31232	106.04587	6.49	430.31097	106.04616	6.19	-3.127	2.727	95.43		
sn-2 MAG (18:1) [a]	$C_{26}H_{42}BNO_4$	444.32797	106.04587	7.43	444.32782	106.04619	7.24	-0.328	3.010	97.39	√	√

sn-2 MAG (19:1) [b]	$C_{27}H_{44}BNO_4$	458.34362	106.04587	8.52	458.34314	106.04618	8.63	-1.038	2.915	101.28		
sn-2 MAG (20:1) [a]	$C_{28}H_{46}BNO_4$	472.35927	106.04587	9.77	472.35944	106.04620	9.96	0.369	3.104	101.99		
sn-2 MAG (21:1) [b]	$C_{29}H_{48}BNO_4$	486.37492	106.04587	11.19	486.37482	106.04615	11.40	-0.197	2.632	95.72		
sn-2 MAG (22:1) [a]	$C_{30}H_{50}BNO_4$	500.39057	106.04587	12.83	500.39011	106.04617	12.94	-0.911	2.821	100.86		√
sn-2 MAG (23:1) [b]	$C_{31}H_{52}BNO_4$	514.40622	106.04587	14.70	514.40704	106.04627	14.42	1.602	3.764	98.10		
sn-2 MAG (24:1) [a]	$C_{32}H_{54}BNO_4$	528.42187	106.04587	16.85	528.42102	106.04616	15.82	-1.601	2.727	93.89		√
sn-2 MAG (25:1) [b]	$C_{33}H_{56}BNO_4$	542.43752	106.04587	19.32	542.43762	106.04618	17.25	0.191	2.915	77.92		
sn-2 MAG (26:1) [b]	$C_{34}H_{58}BNO_4$	556.45317	106.04587	22.14	556.45502	106.04633	18.54	3.331	4.330	73.07		
sn-1 MAG (12:2) [b]	$C_{20}H_{28}BNO_4$	358.21842	106.04587	2.45	358.21844	106.04625	2.44	0.069	3.575	99.71		
sn-1 MAG (15:2) [b]	$C_{23}H_{34}BNO_4$	400.26537	106.04587	3.86	400.26276	106.04621	3.69	-6.510	3.198	95.58		
sn-1 MAG (16:2) [b]	$C_{24}H_{36}BNO_4$	414.28102	106.04587	4.49	414.27881	106.04615	4.98	-5.324	2.632	110.80		
sn-1 MAG (17:2) [b]	$C_{25}H_{38}BNO_4$	428.29667	106.04587	5.23	428.29584	106.04615	5.12	-1.928	2.632	97.85		
sn-1 MAG (18:2) [a]	$C_{26}H_{40}BNO_4$	440.29667	106.04587	6.09	442.31241	106.04619	6.00	0.213	3.010	98.50	√	√
sn-1 MAG (19:2) [a]	$C_{27}H_{42}BNO_4$	456.32797	106.04587	7.09	456.32858	106.04621	7.02	1.346	3.198	98.99		
sn-1 MAG (20:2) [a]	$C_{28}H_{44}BNO_4$	470.34362	106.04587	8.26	470.34451	106.04613	8.23	1.901	2.444	99.69		
sn-1 MAG (22:2) [a]	$C_{30}H_{48}BNO_4$	498.37492	106.04587	11.19	498.37384	106.04610	10.95	-2.159	2.161	97.87		√
sn-1 MAG (24:2) [b]	$C_{32}H_{52}BNO_4$	526.40622	106.04587	15.16	526.40900	106.04609	13.93	5.288	2.067	91.87		
sn-2 MAG (15:2) [b]	$C_{23}H_{34}BNO_4$	400.26537	106.04587	3.64	400.26514	106.04622	3.55	-0.564	3.292	97.56		
sn-2 MAG (16:2) [b]	$C_{24}H_{36}BNO_4$	414.28102	106.04587	4.24	414.28064	106.04622	4.71	-0.907	3.292	111.02		
sn-2 MAG (17:2) [b]	$C_{25}H_{38}BNO_4$	428.29667	106.04587	4.95	428.29944	106.04616	4.89	6.478	2.727	98.86		
sn-2 MAG (18:2) [a]	$C_{26}H_{40}BNO_4$	440.29667	106.04587	5.77	442.31229	106.04617	5.74	-0.058	2.821	99.53	√	√
sn-2 MAG (19:2) [a]	$C_{27}H_{42}BNO_4$	456.32797	106.04587	6.72	456.32898	106.04610	6.71	2.223	2.161	99.80		
sn-2 MAG (20:2) [a]	$C_{28}H_{44}BNO_4$	470.34362	106.04587	7.84	470.34366	106.04616	7.80	0.094	2.727	99.50		√
sn-2 MAG (21:2) [a]	$C_{29}H_{46}BNO_4$	484.35927	106.04587	9.14	484.35855	106.04595	9.20	-1.478	0.746	100.66		
sn-2 MAG (22:2) [a]	$C_{30}H_{48}BNO_4$	498.37492	106.04587	10.66	498.37430	106.04616	10.48	-1.236	2.727	98.35		
sn-2 MAG (23:2) [b]	$C_{31}H_{50}BNO_4$	512.39057	106.04587	12.42	512.38910	106.04617	11.92	-2.861	2.821	95.94		
sn-2 MAG (24:2) [b]	$C_{32}H_{52}BNO_4$	526.40622	106.04587	14.49	526.40417	106.04634	13.37	-3.887	4.424	92.30		

sn-2 MAG (26:2)[b]	$C_{34}H_{56}BNO_4$	554.43752	106.04587	19.69	554.44281	106.04617	16.20	9.548	2.821	82.28		
sn-1 MAG (16:3)[b]	$C_{24}H_{34}BNO_4$	412.26537	106.04587	3.78	412.26056	106.04620	4.05	-11.657	3.104	107.26		
sn-1 MAG (18:3)[a]	$C_{26}H_{38}BNO_4$	440.29667	106.04587	5.05	440.29614	106.04613	5.05	-1.194	2.444	100.03		
sn-1 MAG (20:3)[a]	$C_{28}H_{42}BNO_4$	468.32797	106.04587	6.75	468.32809	106.04620	6.67	0.265	3.104	98.82		√
sn-1 MAG (21:3)[b]	$C_{29}H_{44}BNO_4$	482.34362	106.04587	7.80	482.34988	106.04607	7.98	0.811	1.878	102.31		
sn-1 MAG (22:3)[a]	$C_{30}H_{46}BNO_4$	496.35927	106.04587	9.02	496.35934	106.04615	8.94	0.149	2.632	99.07		
sn-1 MAG (24:3)[b]	$C_{32}H_{50}BNO_4$	524.39057	106.04587	12.06	524.38837	106.04611	11.91	-4.188	2.255	98.76		
sn-2 MAG (11:3)[b]	$C_{19}H_{24}BNO_4$	342.18712	106.04587	1.78	342.18338	106.04620	2.22	-10.916	3.104	124.72		
sn-2 MAG (12:3)[b]	$C_{20}H_{26}BNO_4$	356.20277	106.04587	2.05	356.20258	106.04616	2.27	-0.520	2.727	110.73		
sn-2 MAG (16:3)[b]	$C_{24}H_{34}BNO_4$	412.26537	106.04587	3.64	412.26434	106.04614	3.90	-2.488	2.538	107.14		
sn-2 MAG (18:3)[a]	$C_{26}H_{38}BNO_4$	440.29667	106.04587	4.84	440.29636	106.04613	4.83	-0.694	2.444	99.79		√
sn-2 MAG (19:3)[b]	$C_{27}H_{40}BNO_4$	454.31232	106.04587	5.58	454.31110	106.04615	5.88	-2.676	2.632	105.38		
sn-2 MAG (20:3)[a]	$C_{28}H_{42}BNO_4$	468.32797	106.04587	6.44	468.32797	106.04617	6.36	0.009	2.821	98.76	√	√
sn-2 MAG (21:3)[b]	$C_{29}H_{44}BNO_4$	482.34362	106.04587	7.42	482.34695	106.04616	7.66	-5.264	2.727	103.23		
sn-2 MAG (22:3)[a]	$C_{30}H_{46}BNO_4$	496.35927	106.04587	8.56	496.35919	106.04619	8.51	-0.153	3.010	99.42		
sn-2 MAG (23:3)[b]	$C_{31}H_{48}BNO_4$	510.37492	106.04587	9.88	510.37448	106.04613	10.37	-0.854	2.444	104.96		
sn-2 MAG (24:3)[b]	$C_{32}H_{50}BNO_4$	524.39057	106.04587	11.40	524.39075	106.04626	11.43	0.351	3.670	100.26		
sn-1 MAG (19:4)[b]	$C_{27}H_{38}BNO_4$	452.29667	106.04587	4.92	452.30103	106.04610	4.97	9.649	2.161	101.03		
sn-1 MAG (20:4)[a]	$C_{28}H_{40}BNO_4$	466.31232	106.04587	5.69	466.31241	106.04614	5.78	0.202	2.538	101.58	√	√
sn-1 MAG (21:4)[b]	$C_{29}H_{42}BNO_4$	480.32797	106.04587	6.57	480.32291	106.04611	6.52	-10.526	2.255	99.24		
sn-1 MAG (22:4)[a]	$C_{30}H_{44}BNO_4$	494.34362	106.04587	7.60	494.34708	106.04610	7.47	7.008	2.161	98.29		
sn-1 MAG (24:4)[b]	$C_{32}H_{48}BNO_4$	522.37492	106.04587	10.16	522.37152	106.04632	9.77	-6.501	4.235	96.16		
sn-2 MAG (15:4)[b]	$C_{23}H_{30}BNO_4$	396.23407	106.04587	2.69	396.23178	106.04626	2.56	-5.768	3.670	95.19		
sn-2 MAG (16:4)[b]	$C_{24}H_{32}BNO_4$	410.24972	106.04587	3.10	410.25217	106.04614	3.28	5.983	2.538	105.82		
sn-2 MAG (18:4)[a]	$C_{26}H_{36}BNO_4$	438.28102	106.04587	4.12	438.28180	106.04617	4.04	1.790	2.821	98.06		
sn-2 MAG (20:4)[a]	$C_{28}H_{40}BNO_4$	466.31232	106.04587	5.47	466.31232	106.04617	5.53	0.009	2.821	101.10	√	√
sn-2 MAG (21:4)[b]	$C_{29}H_{42}BNO_4$	480.32797	106.04587	6.30	480.32623	106.04610	6.25	-3.614	2.161	99.21		

sn-2 MAG (22:4) [a]	$C_{30}H_{44}BNO_4$	494.34362	106.04587	7.27	494.34366	106.04618	7.15	0.089	2.915	98.35		
sn-2 MAG (24:4) [b]	$C_{32}H_{48}BNO_4$	522.37492	106.04587	9.65	522.37518	106.04620	9.31	0.505	3.104	96.48		
sn-1 MAG (20:5) [a]	$C_{28}H_{38}BNO_4$	464.29667	106.04587	4.72	464.30164	106.04613	4.77	10.714	2.444	101.06		
sn-1 MAG (22:5) [a]	$C_{30}H_{42}BNO_4$	492.32797	106.04587	6.79	492.32602	106.04620	6.69	-3.952	3.104	98.53		√
sn-1 MAG (25:5) [b]	$C_{33}H_{48}BNO_4$	534.37492	106.04587	11.69	534.37421	106.04630	10.78	-1.321	4.047	95.38		
sn-2 MAG (12:5) [b]	$C_{20}H_{22}BNO_4$	352.17147	106.04587	1.10	352.17365	106.04610	2.05	6.204	2.161	186.36		
sn-2 MAG (20:5) [a]	$C_{28}H_{38}BNO_4$	464.29667	106.04587	4.57	464.29764	106.04617	4.59	-0.742	2.821	100.44	√	√
sn-2 MAG (21:5) [b]	$C_{29}H_{40}BNO_4$	478.31232	106.04587	5.46	478.31454	106.04613	5.13	4.650	2.444	93.96		
sn-2 MAG (22:5) [a]	$C_{30}H_{42}BNO_4$	492.32797	106.04587	6.52	492.32877	106.04615	6.42	-1.045	2.632	98.47	√	√
sn-2 MAG (24:5) [b]	$C_{32}H_{46}BNO_4$	520.35927	106.04587	9.32	520.36383	106.04628	8.90	8.771	3.858	95.49		
sn-1 MAG (22:6) [a]	$C_{30}H_{40}BNO_4$	490.31232	106.04587	5.48	490.31201	106.04614	5.47	-0.624	2.538	99.82		√
sn-1 MAG (24:6) [b]	$C_{32}H_{44}BNO_4$	518.34362	106.04587	7.93	518.34344	106.04617	6.81	-0.339	2.821	85.88		
sn-2 MAG (18:6) [b]	$C_{26}H_{32}BNO_4$	434.24972	106.04587	2.55	434.25412	106.04620	2.98	10.143	3.104	116.86		
sn-2 MAG (19:6) [b]	$C_{27}H_{34}BNO_4$	448.26537	106.04587	3.05	448.25980	106.04617	3.51	-12.416	2.821	115.08		
sn-2 MAG (22:6) [a]	$C_{30}H_{40}BNO_4$	490.31232	106.04587	5.27	490.31265	106.04618	5.26	0.682	2.915	99.81	√	√
sn-2 MAG (24:6) [b]	$C_{32}H_{44}BNO_4$	518.34362	106.04587	7.57	518.34381	106.04613	6.56	0.375	2.444	86.66		√

[a] MAG chemical standard, including commercial and synthesized compounds.

[b] predicted MAG species in biological samples.

√, recorded in the database.
